# Translational profiling identifies sex-specific metabolic and epigenetic reprogramming of cortical microglia/macrophages in APPPS1-21 mice with an antibiotic-perturbed-microbiome

**DOI:** 10.1186/s13024-023-00668-7

**Published:** 2023-12-16

**Authors:** Shabana M. Shaik, Yajun Cao, Joseph V. Gogola, Hemraj B. Dodiya, Xulun Zhang, Hejer Boutej, Weinong Han, Jasna Kriz, Sangram S. Sisodia

**Affiliations:** 1 Dept. of Neurobiology, The University of Chicago, Chicago, IL USA; 2 CERVO Brain Research Centre and Department of Psychiatry and Neuroscience, Laval University, Québec, QC Canada

**Keywords:** Alzheimer’s disease, Microglia, Macrophage, Microbiome, Proteomics, Metabolism, Epigenetics, Inflammation, Sex, Translating ribosome affinity purification

## Abstract

**Background:**

Microglia, the brain-resident macrophages perform immune surveillance and engage with pathological processes resulting in phenotype changes necessary for maintaining homeostasis. In preceding studies, we showed that antibiotic-induced perturbations of the gut microbiome of APPPS1-21 mice resulted in significant attenuation in Aβ amyloidosis and altered microglial phenotypes that are specific to male mice. The molecular events underlying microglial phenotypic transitions remain unclear. Here, by generating ‘APPPS1-21-CD11br’ reporter mice, we investigated the translational state of microglial/macrophage ribosomes during their phenotypic transition and in a sex-specific manner.

**Methods:**

Six groups of mice that included WT-CD11br, antibiotic (ABX) or vehicle-treated APPPS1-21-CD11br males and females were sacrificed at 7-weeks of age (*n* = 15/group) and used for immunoprecipitation of microglial/macrophage polysomes from cortical homogenates using anti-FLAG antibody. Liquid chromatography coupled to tandem mass spectrometry and label-free quantification was used to identify newly synthesized peptides isolated from polysomes.

**Results:**

We show that ABX-treatment leads to decreased Aβ levels in male APPPS1-21-CD11br mice with no significant changes in females. We identified microglial/macrophage polypeptides involved in mitochondrial dysfunction and altered calcium signaling that are associated with Aβ-induced oxidative stress. Notably, female mice also showed downregulation of newly-synthesized ribosomal proteins. Furthermore, male mice showed an increase in newly-synthesized polypeptides involved in FcγR-mediated phagocytosis, while females showed an increase in newly-synthesized polypeptides responsible for actin organization associated with microglial activation. Next, we show that ABX-treatment resulted in substantial remodeling of the epigenetic landscape, leading to a metabolic shift that accommodates the increased bioenergetic and biosynthetic demands associated with microglial polarization in a sex-specific manner. While microglia in ABX-treated male mice exhibited a metabolic shift towards a neuroprotective phenotype that promotes Aβ clearance, microglia in ABX-treated female mice exhibited loss of energy homeostasis due to persistent mitochondrial dysfunction and impaired lysosomal clearance that was associated with inflammatory phenotypes.

**Conclusions:**

Our studies provide the first snapshot of the translational state of microglial/macrophage cells in a mouse model of Aβ amyloidosis that was subject to ABX treatment. ABX-mediated changes resulted in metabolic reprogramming of microglial phenotypes to modulate immune responses and amyloid clearance in a sex-specific manner. This microglial plasticity to support neuro-energetic homeostasis for its function based on sex paves the path for therapeutic modulation of immunometabolism for neurodegeneration.

**Supplementary Information:**

The online version contains supplementary material available at 10.1186/s13024-023-00668-7.

## Background

The cardinal pathological lesions found in the brains of patients with Alzheimer’s (AD) are the accumulation of extracellular deposits of small, ~ 4 kDa amyloid beta (Aβ) peptides, and intracellular accumulation of hyperphosphorylated tau, that are linked with neurodegeneration and ultimately, neuronal death [1]. Inflammation associated with amyloid deposition reflects the activation of a subset of astrocytes and microglia that adopt pro-inflammatory phenotypes [2]. While sex-specific differences in pathology are not evident in the brains of AD patients, clinical studies reveal that females show more prevalence and progression of AD compared with males [3]. Sex has a crucial role in the incidence of neurological diseases and sex-specific genetic architecture is thought to be driven through the organizational effects during differentiation and development of the brain [4–6]. Sex-biased gene expression likely regulates sex-biased protein synthesis in response to various cellular or environmental cues thus influencing sex-specific aging or progression of disease. For example, the ε4 allele of the apolipoprotein E gene (*APOE4*) is a significantly greater AD risk factor for females than for males [7, 8]. Sex differences are often linked with the primary sex hormone in females, the estrogen 17β-estradiol, and age-related depletion of estrogen is associated with increased risk of AD [9, 10]. However, the biological underpinnings of the associated sexual dimorphism are not fully understood.

Among the brain-resident macrophages that maintain tissue homeostasis, microglia represent major population of immune cells in brain parenchyma whereas non-parenchymal CNS-associated macrophages (CAMs) or border-associated macrophages are localized in the brain-circulation interface [11]. As sentinel cells, microglia constantly screen the brain parenchyma and engage in pathological processes by changing their morphology, differentially expressing various antigens, and become phagocytic [12]. This activation causes release of a wave of chemical mediators including cytokines, chemokines, reactive oxygen, and nitrogen species that promote the neuroinflammatory milieu [13]. Thus, microglial homeostasis represents a highly plastic multifaceted response, finely tuned by the nature of the stimulus and the molecular repertoire involved. Microglia exhibit unique spatio-temporal gene expression profiles and express an array of cellular receptors [14] and signaling molecules referred to as “sensome” [15]. They detect both endogenous stimuli such as aggregated proteins or cell damage and exogenous peripheral stimuli like pathogenic molecules and respond by undergoing polarization to distinct phenotypes [12]. These cells can switch from an ‘M0’ homeostatic phenotype to a neurodegenerative phenotype (MGnD) [16] or ‘dark’ microglia during the course of aging and disease [17–19]. Whether the phenotypical transformation of microglial cells is a neuroprotective response to cellular damage at early stages or are principal drivers of disease remains an intriguing question.

Host commensal gut microbes are shown to be crucial for microglial maturation, and function that involve innate immune responses to promote the maintenance of microglia under steady state conditions [20]. Microglia from germ-free mice exhibit different transcriptomic profiles and antibiotic (ABX)-treatment of specific pathogen-free (SPF) mice revealed sexually biased microglial responses that occur in a temporal manner [21]. In our preceding efforts, we demonstrated a strong connection between the gut microbiome and Aβ amyloidosis and altered microglial phenotypes in mouse models that express familial AD-linked variants of APP and PS1 [22–24]. Specifically, ABX-mediated alterations in gut microbiome of APPswe/PS1ΔE9 [25] and APPPS1-21 [26] mice models resulted in significant attenuation of Aβ amyloidosis that occurs only in male mice [22–24]. Transcriptomic profiling of cortical RNAs from 7-weeks old APPPS1-21 mice treated with ABX revealed ‘M0’ microglial signatures in male mice while the females showed persistent ‘MGnD’ signatures [24]. Further, we also showed that short-time, postnatal ABX-treatment had similar effect on Aβ deposition, microglial phenotypes, and cerebral cortex transcriptomic profiles based on sex [27]. The mechanism(s) underlying the sex-specific clearance of Aβ mediated by ABX-treatment induced changes in gut microbiome remains unclear.

High-throughput transcriptome profiling has been a powerful tool for the characterization of microglial phenotypes in normal and diseased states [28–31]. However, there is a large gap in understanding how the transcriptional profiles of microglia relate to the functional protein abundance due to lack of models that allow reliable in vivo proteomics. Classically, the proteome of CD11b + microglia has been investigated using flow cytometry (FACS) or magnetic bead based (MACS) cell sorting [32, 33]. Alternative approaches like RiboTag and Translating ribosome affinity purification (TRAP) [34] combine cell-type specific transgene expression with affinity purification of translating ribosomes thereby enabling the profiling of cell-type mRNA. RiboTag strategy involves the expression of hemagglutinin (HA) tagged ribosomal protein RPL22 under Cx3cr1 promoter and was used to establish RiboTag profiling as a valuable tool to investigate microglia [35]. TRAP approach involves expression of EGFP-tagged ribosomal protein Rpl10a transgene under the control of CD11b promoter. Using a modified TRAP model (CD11brGFP mice) for analysis of translational state of microglial ribosomes, a marked dissociation of microglial mRNA and protein networks following innate immune challenge has been reported [36].

To understand the molecular mechanism(s) underlying the ABX-mediated, sex-specific Aβ clearance and the observed microglial phenotypes, we employed CD11brGFP mice to generate ‘APPPS1-21-CD11br’ transgenic mice. We first validated the mice model by analyzing the mRNAs reported to be expressed by CD11b + macrophage/microglia and then assessed their protein networks. Confirming our earlier findings, we report that ABX-treatment resulted in decreased Aβ burden in male APPPS1-21-CD11br mice. Quantitative profiling of newly synthesized peptides was performed by immunoprecipitation of cortical homogenates of WT-CD11br and APPPS1-21-CD11br male and female mice treated with ABX or vehicle. We first assessed sex-specific microglial/macrophage protein networks in order to provide insights into global changes that occur due to Aβ-induced microglial activation and then due to changes mediated by ABX-treatment. Analysis of peptides in ABX-treated mice revealed metabolic and epigenetic reprogramming of microglial translational landscapes associated with their function that are sex-specific. This shift mediates neuroprotective phenotypes associated with phagocytic clearance of Aβ in male mice but persistent mitochondrial dysfunction and inflammatory phenotype in female mice. To validate the key proteins identified by the translatome, we captured polysomes from CD11b + cells and analyzed the expression of the corresponding mRNAs by qPCR.

To understand the impact of ABX-induced changes in gut metabolites that can modulate neuro-immune responses we carried out metabolomic profiling of the cecal contents from vehicle- or ABX-treated APPPS1-21-CD11br mice. We find that ABX-treated mice have altered levels of short chain fatty acids (SCFAs) and metabolites of tryptophan catabolism that can support a shift in metabolism for microglial activation to modulate neuro-immune responses. Taken together, our proteomic dataset represents the first comprehensive analysis of the dynamic translational state of microglial ribosomes and, in conjunction with targeted metabolomic analyses, provides molecular insights into the effect of microbiome on phenotypic changes associated with sex-specific microglial activation.

## Methods

### Transgenic mice generation, genotyping and handling

CD11br mice [36] and APPPS1-21 [26] mouse lines were maintained on a C57BL/6Cj background and crossed to generate APPPS1-21-CD11br transgenic mice. The transgenic mice were genotyped by PCR amplification for the APP, PS1 and EGFP gene using genomic DNA isolated from the tail samples of all the mice used in the study. A 500 bp APP fragment was amplified using FP (5’ CGA CAG TGA TCG TCA TCA CCT 3’) and RP (5’ CTT AGG CAA GAG AAG CAG CTG 3’) and 300 bp for PS1-FP (5’ CAG GTG CTA TAA GGT CAT CC 3’) and RP (5’ ATC ACA GCC AAG ATG AGC CA 3’). 329 bp EGFP fragment was amplified using FP (5’AGTTCA TCTGCACCACCGGC 3’) and RP (5’-CGGCCATGATATAGACGTTG-3’). Mice were housed in sterile cages and fed ad libitum on standard chow.

### Antibiotic (ABX)-treatment regime

Pups receiving the ABX were gavaged (200 μl ABX using animal feeding needles; catalog number 7901; Cadence) from P14-P21 followed by an ad libitum access to freshly prepared 1:50 diluted ABX water until the time of sacrifice according to our established protocol [24]. Briefly, ABX cocktail (4 mg/ml kanamycin (Sigma-Aldrich, K4000-5 g), 0.35 mg/ml gentamicin (Sigma-Aldrich G1914), 8,500 U/ml colistin (Sigma- Aldrich C4461), 2.15 mg/ml metronidazole (Sigma-Aldrich, M1547), 0.45 mg/ml vancomycin (Sigma-Aldrich V2002) was prepared in autoclaved water. During the week of postnatal ABX gavage, all mice were transferred to a new sterile cage after each gavage. Parents from the same cage as pups receiving ABX treatment were euthanized after weaning the pups and were not used for any breeding or future experiments. Water with ABX was changed once every week. Water gavaged pups were used as vehicle-treatment.

### Necropsy and tissue harvesting

The necropsy was performed according to procedures approved by IACUC. Briefly, a mixture of ketamine and xylazine was introduced i.p., and after confirmation of anesthesia, the heart was accessed through abdominal surgery, and blood was collected from the right ventricle by using a 25-gauge needle and stored in buffered sodium citrate blood collection tubes (catalog number 366393; BD Vacutainer) on ice. After the blood collection, mice were perfused by using cold for Hank’s balanced salt solution (HBSS) (Gibco, Cat. No 14155) for 3 min. Brains were then excised and dissected into two hemispheres (one hemisphere was post-fixed with 4% paraformaldehyde, and the other was frozen for proteomic studies). Cecum was removed from small and large intestine, weighed, and stored at − 80 °C for further investigations.

### Immunocytochemistry

The immunofluorescence staining was performed to evaluate Aβ amyloidosis as per the published procedure [24]. A full series of 40 mm thick brain sections were utilized for Aβ (mouse anti-**A**β; 3D6; 1:10,000) staining. Briefly, free-floating level-matched sections were washed with dilution media for 60 min (10 min/wash). Sections were then incubated in serum blocking solution for 1 h at room temperature, followed by primary antibody incubations for overnight in a 4 °C cold room. The next day, sections were washed with dilution media for 60 min (10 min/wash), followed by secondary antibody incubation at room temperature for 1 h. We used donkey anti-mouse IgG H&L (Alexa Fluor® 555) (ab150106; Invitrogen, 1:500) as secondary antibody. Sections were then washed with dilution media and mounted on glass slides followed by cover slipping using fluoromount aqueous mounting medium (Sigma-Aldrich; F4680). 3D6 + Aβ plaque images were captured by using 3D Histech Pannoramic MIDI whole slide scanner with a Zeiss AxioCam MRm CCD camera by the University of Chicago Integrated Light Microscopy Facility personnel.

We performed immunofluorescence staining using primary antibodies specific for microglia including P2RY12 (rabbit anti-P2RY12; HPA014518; Sigma 1:500), TMEM119 (rabbit anti-TMEM119; abcam ab209064) and CD11b (rat anti-CD11b; abcam ab8878) to show the enrichment of microglia in CD11b + cells. These antibodies were followed by incubation using donkey anti-rabbit IgG Alexa Fluor® 594 (ab150076; 1:500), and Alexa Fluor® 647 (abcam, ab 150,075; 1:500) and goat anti-rat IgG Alexa Fluor® 488 (abcam, ab150157; 1:500) secondary antibodies, respectively. We performed double label immunofluorescence staining using primary antibodies specific for macrophages/microglia (rabbit anti-IBA1; 019–19741; Wako; 1:500) and GFP (goat anti-GFP; ab5450; Abcam; 1:200) to confirm the transgene expression in our model. These antibodies were followed by secondary antibody incubation as mentioned above using donkey anti-rabbit IgG H&L (Alexa Fluor® 647) (ab150075; 1:500) and donkey anti-goat IgG H&L (Alexa Fluor® 488) (ab150129; 1:500), respectively.

### Imaging and processing

Images were captured on a SP8 Laser Scanning Confocal microscope running LAS_X image collection software (Leica) at the University of Chicago Light Microscopy Core Facility with a 40 × plan-apochromat 1.25 numerical aperture (NA) oil-immersion objective. Digital spectral windows for detection were set based on common emission spectra for the associated fluorophores, with at least 10 nm spacing between spectra. Low-magnification survey images were captured at a digital zoom of 2.0x, and higher-magnification images at a digital zoom of 4.0x. All samples were imaged with identical collection parameters, collected at 1024 × 1024-pixel density with a line average setting of 16, and z-stacks at a step-size of 1 μm. Imaging data were exported as single channel tiff stacks for further processing. Z-stacks were flattened using the two-dimensional maximum intensity projection algorithm in ImageJ (NIH), background subtracted with a 50-pixel radius rolling ball setting, pseudo-colored, overlayed, and exported as tiff files before compiling into figures.

### Aβ burden analysis

Aβ burden and amyloid plaque size were evaluated on slide scanner images of 3D6 + immunoreactive structures as per the established protocol [24]. Total of six sections at an equidistance of 480 μm was used. Using Fiji Image J software (NIH, Image J 1.51n), average plaque size and Aβ fractional area (amyloid burden) were generated. Individual images were created with the 3D Histech Pannoramic viewer software (3DHistech Kft). Images were then normalized, and an automated thresholding based on entropy of the histogram was used to identify the amyloid plaques. After converting images into 8-bit format, specific threshold number was applied which was then followed by “fill holes” and “watershed” algorithms for a binary conversion. Finally, plaque number, and total area occupied by plaque (i.e., Aβ burden) was calculated using the “analyze particles”. Graphs were plotted for amyloid burden.

### Western blot

Each hemicortex was weighed and homogenized on ice in 8 volume of lysis buffer (50 mM Tris, pH7.4, 150 mM NaCl, 5 mM EDTA, 0.5% NP-40, and 0.5% sodium deoxycholate) using a Kontes homogenizer. The homogenates were centrifuged at 16,000 g for 20 min at 4 °C, and the supernatant fractions were saved as the detergent-soluble lysates. 100 μg of the detergent-insoluble lysates was resolved on a Tris-tricine gel or on a 10% SDS-PAGE to detect APP fragments, PS1 fragments and β-actin. 6E10 (Bio legend) was used to detect human specific full-length APP, soluble APPα, CTFβ, while Aβ. C1/6.1 (Biolegend) was used to detect both mouse and human full-length APP and CTFα and CTFβ. PS1 NT1 (Biolegend) was used to detect PS1, and β-actin antibody (Genscript) was used to detect β-actin.

### Cortical RNA isolation and RT-PCR analysis

Brain cortex was dissected from the frozen hemibrains of WT and APPPS1-21-CD11br mice treated with vehicle or ABX along with APPPS1-21 and non-transgenic mice followed by homogenization in Trizol reagent (Ambion). 0.5 μg of total RNA was used for cDNA synthesis using SuperScript IV VILO MasterMix with ezDNAse enzyme (Invitrogen) followed by PCR with EGFP forward (FP) and reverse (RP) primers using Taq DNA Pol (New England Biolabs). Gapdh was used as a reference gene. The amplified products were fractionated on 1% agarose gels and stained with ethidium bromide.

### TRAP protocol and purification of mRNA from Flag-EGFP-RPL10a

We used the modified TRAP protocol as described previously [36]. Briefly, brain cortex samples were placed into ice-cold dissection buffer followed by a homogenization (10% w/v) in tissue lysis buffer. Samples were then centrifuged at 2,000 g for 10 min at 4 C. 1/9 sample volume of 10% NP-40 and 300 mM DHPC were added to the supernatant fractions. Samples were then incubated for 30 min at 4 °C on a tube rotator followed by centrifugation at 20,000 g for 10 min at 4 °C. Each supernatant fraction was added to anti-FLAG agarose affinity resin and incubated overnight at 4 °C on a rotator. The following day, the beads were recovered by centrifugation and washed 3 times with high-salt buffer (20 mM HEPES–KOH pH 7.3, 200 mM KCl, 12 mM MgCl2, 1% NP-40, 0.5 mM DTT, and 100 mg/mL cycloheximide). The bead pellet was used for mRNA purification by resuspending the bead pellet in 100 μL of Nanoprep lysis buffer with β -mercaptoethanol and incubated at room temperature for 10 min. The RNA purification was performed according to the manufacturer’s instructions (Stratagene, Absolutely RNA Nanoprep kit). 2 biological replicates were used for each group. The purified RNA was used for cDNA synthesis followed by RT-PCR or qPCR analysis.

### RT-(q)PCR analysis

Quantity of purified ribosome-associated mRNA was measured using a NanoDrop ND-1000 Spectrophotometer (Nanodrop Technologies, USA). 50 ng of RNA purified from pull-down was used for cDNA synthesis with dT18 oligo primer using Superscript VILO Master Mix. This cDNA was used for all the RT-PCR and qPCR assays using specific primer sets. EGFP FP and RP primer set was used for RT-PCR using Taq DNA Polymerase (New England Biolabs) to show the expression of FLAG-EGFP-Rpl10a transgene. qPCR was performed on QuantStudio 3 Real Time PCR (Thermofisher) using Power SYBR Green Master Mix according to the manufacturer’s instructions (Applied Biosystems). A melting curve was performed to assess non- specific signal. Glyceraldehyde-3-phosphate dehydrogenase (*GAPDH*) was used as a reference gene. For expression analysis 2^^ΔCT^ method was used wherein the difference between C_T_ values of the gene interest and reference gene is calculated for all the samples. UD refers to undetermined. GraphPad Prism was used to plot the 2^^ΔCT^ values shown as bar diagram. Error bars represent SD of three technical replicates. To confirm the microglia specific transcripts, cDNA was used for PCR with primer sets to amplify *IbaI*, *P2ry12*, *Tmem119*, *Cx3cr1*, and *Trem2*. Primers for *Gfap*, *Tubb3* and *Cldn11* were used as markers for astrocytes, neurons, and oligodendrocytes. The list of all the primers is presented in Supplemental Table 3.

### TRAP protocol and purification of peptides from FLAG-EGFP-RPL10A

For purification of peptides, three half cortices were pooled per sample for a total of 15 brains. For ABX and vehicle-treated groups 3 samples with similar amyloid burdens were pooled. Following TRAP protocol, the beads pellet was used for peptide purification. For this, bead pellets were resuspended in EDTA-elution buffer (10 mM HEPES–KOH pH 7.3, 150 mM KCl, 5 mM MgCl2, 20 mM EDTA, and protease inhibitor cocktail) and incubated for 30 min at room temperature on a tube rotator. The eluate was recovered by centrifugation at 7,000 rpm for 15 min. Collected ribosome-associated peptides were sequenced by mass spectrometry using Orbitrap fusion mass spectrometer.

#### Additional note

An estimate of the total number of microglia reported in the adult mouse brain is 3.5 × 10^6^, and vary from 5% of the total cells in the cortex and corpus callosum to 12% in the substantia nigra [37]. Considering the above, half cortex is composed of 87,500 microglia ((3.5 × 10^6^)/2 × 5/100) and assuming a modest expression of 100 copies of the EGFP-FLAG-Rpl10a per cell, this equals to 1.45 × 10^–17^ mol (87,500 × 100/6.023 × 10^23^) or 0.8 pg (1.45 × 10^–17^ × 53,000) of the fusion protein which doesn’t fall within the detection range using Western blot. For Fig. 2D, we performed the western blot analysis to establish and validate the pull-down of newly synthesized peptides from microglia using whole brain lysates from two WT-CD11br male mice aged 7-weeks.

### Sample preparation prior to mass spectrometry

Each sample was concentrated using a 3 kDa Amicon Ultra-0.5 Centrifugal Filter device (Millipore) by 10 min centrifugation at 14,000 × *g* followed by a wash with 500 μL of 50 mM ammonium bicarbonate and centrifugation in the same conditions. For each sample the volume was adjusted to 50 μL with 50 mM ammonium bicarbonate and sodium deoxycholate was added to a final concentration of 1%. Protein denaturation was performed by heating at 95 °C for 5 min. Reduction and alkylation of cysteine disulfide bridges were performed by addition of 1,4 dithiothreitol (DTT) (final concentration 0.2 mM) and incubation at 37 °C for 30 min followed by addition of iodoacetamide (final concentration 0.8 mM) and incubation at 37 °C for 30 min in the dark. 200 ng of trypsin was then added, and samples were incubated at 37 °C overnight for proteolysis. Enzymatic digestion was terminated by acidification using 60 μL of 3% acetonitrile, 1% trifluoroacetic acid, 0.5% acetic acid. The peptides resulting from trypsin digestion were purified on StageTip according to Rappsilber et al. [38] using C18 Empore reverse phase (CDS). The samples were vacuum dried and stored at -20 °C prior to mass spectrometry analysis.

### LC–MS/MS analysis

Each sample was resuspended at 0.2 μg/μL with 2% acetonitrile, 0.05% TFA and peptide concentration was determined using absorbance measurement at 205 nm with Nanodrop (Thermo Fisher Scientific). 1 μg of each sample was then analyzed by liquid chromatography coupled to tandem mass spectrometry (LC-MSMS) using an U3000 RSLCnano chromatographic system (Thermo Fisher Scientific) interfaced with an Orbitrap Fusion mass spectrometer (Thermo Fisher Scientific). The chromatographic separation was performed on a reverse phase Acclaim PepMap 100 C18 column (75 μm internal diameter, 3 μm particles and 500 mm length) (Thermo Fisher Scientific) using a 5–45% solvent B in 90 min gradient (Solvent A: 5% acetonitrile, 0.1% formic acid; solvent B: 80% acetonitrile, 0.1% formic acid) with a flow rate of 300 nl/min while the mass spectrometer was operating in Data Dependent Acquisition mode using Thermo XCalibur software version 3.0.63. Full scan mass spectra (350 to 1800 m/z) were acquired in the orbitrap at a resolution of 120 000. Internal calibration using lock mass on the m/z 445.12003 siloxane ion was used. Each MS scan was followed by acquisition of fragmentation MSMS spectra of the most intense ions for a total cycle time of 3 s (top speed mode). The selected ions were isolated using the quadrupole analyzer in a window of 1.6 m/z and fragmented by Higher energy Collision induced Dissociation (HCD) with 35% of collision energy. The resulting fragments were detected by the linear ion trap in Rapid scan rate. Dynamic exclusion of previously fragmented peptides was set for a period of 20 s and a tolerance of 10 ppm.

### Label free quantification

Spectra were searched against a mouse protein database (Uniprot Mus musculus Reference Proteome – UP000000589 – 2019/06/11) using the Andromeda module of MaxQuant software v.1.6.10.43 [39]. Trypsin/P enzyme parameter was selected with two possible missed cleavages. Carbamidomethylation of cysteines was set as fixed modification, methionine oxidation and acetylation of protein N-terminus as variable modifications. Mass search tolerance were 4.5 ppm and 0.6 Da for MS and MS/MS respectively. For validation of identifications, a maximum False Discovery Rate of 0.01 at PSM (Peptide Spectrum Match) and protein levels was used based on a target/decoy search. MaxQuant was also used for Label Free Quantification. The ‘match between runs’ option was used with 20 min as alignment time window and 0.7 min as match time window values. Only unique and razor peptides were used for quantification. All other parameters were set at default values.

### Data treatment and statistical analysis related to proteomics

The proteinGroups.txt file generated by MaxQuant was used in R software v 3.4 (R Development Core Team 2005, http://www.R-project.org) to perform the following steps. The LFQ intensity values of each protein in each sample were normalized using the median of all LFQ intensity values in each sample. Missing values in the dataset were imputed using a noise value calculated as the first centile of all LFQ intensity values of each sample. This dataset was used to generate a Principal Component Analysis and a Heatmap with hierarchical clustering in R software. Pairwise analyses were then performed to identify differentially expressed proteins between 2 groups of samples. Only proteins having at least 80% of LFQ intensity values, before missing value imputation, in one of the two groups to compare were considered as quantifiable and only proteins with at least 2 quantified peptides were kept for further analysis. For each protein, a ratio between the two conditions to compare was calculated using the average of protein intensities in all samples of the same group. These ratios were then converted into z-score (*z* = (*x*-*μ*)/*σ* were (*x* = log2(ratio); *μ* = average of all log2(ratios); *σ* = standard deviation of all log2(ratios)) for data centering. A Limma statistical test [40] was finally performed to determine the probability of variation (*p*-value) of each protein between the two groups. The Benjamini-Hochberg method was used to adjust the *p*-values for multiple testing and thus obtain *q*-values. Proteins with a *q*-value < 0.05 and absolute value of *z*-score |*z*|> 1.96 were considered as significantly differentially expressed between the two groups of samples.

#### Note

One sample per group has been removed following the analysis of all samples.

### Cytoscape analysis

Data from mass spectrometry was analyzed with ClueGo [41] and CluePedia [42] applications (version 2.5.7) using the Cytoscape environment (3.8.2). Differentially expressed proteins (DEPs) (FDR < 0.05) from each comparison (RM-TMV; RF-TFV; TMV-TMA; TFV-TFA) was used to generate biological networks using different ontology sources like the Gene Ontology (GO), (terms included-Biological Process, Molecular Function, Cellular component, Immune System Process), Kyoto Encyclopedia of Genes and Genomes (KEGG) and Reactome Pathways. The GO interval was between 3 (Min level) and 8 (Max level). The Kappa score threshold was 0.4. For the enrichment of biological terms and groups, we used the two-sided (Enrichment/Depletion) tests based on the hypergeometric distribution. We set the statistical significance to 0.05 and we used the Benjamini–Hochberg adjustment to correct the *p*-value for the terms and the groups created by ClueGo. CluePedia was used to represent functionally grouped network with pathways and genes as nodes linked based on their kappa score (> 0.3). Terms and their associated genes share the color and only the label of the most significant term per group is shown. The leading group term is based on %genes/term vs cluster. GO/pathway terms in the network are labeled in black. Upregulated and downregulated DEPs are shown in red and blue respectively. %Term per group is represented in pie chart and the bar charts in SI represent the number of genes associated with the significant parent term (%genes/term).

### Protein–protein interactions (PPI) networks

The PPI networks for differentially expressed proteins identified from each pair-wise comparison were retrieved using the search tool of interacting protein database of STRING v1.5 [43]. Given the list of proteins as input STRING integrates both known and predicted PPIs to predict functional interaction of proteins. Active interaction sources including text mining, experiments, databases, and co-expression as well as species limited to ‘*Mus musculus*’ with an interaction score > 0.4 were applied to construct the PPI networks. Cytoscape version was used to visualize the PPI network. In the networks, the nodes represent proteins, and the edges represent the interactions. Singlets or proteins with no interacting partners are not shown in the network. Upregulated proteins are shown in red and downregulated in blue. Highly connected clusters or subnetworks within the PPI network were identified using the MCODE [44] (Molecular Complex Detection) module in Cytoscape. The significant clusters were identified with parameters that included degree cutoff 2 and node score cutoff 0.2 with no fluff.

### Functional enrichment analysis

To functionally characterize the cluster, StringApp was used to perform functional enrichment analysis to identify statistically significant terms that span GO Biological Process, Molecular Function, Cellular Component, KEGG and Reactome pathways. Terms associated with upregulated proteins are shown in red and downregulated in blue. A right-side hypergeometric test was used for calculation of *p*-value followed by multiple test correlation by Benjamini–Hochberg adjustment and pathways with adjusted *p*-value < 0.05 are considered for significance. We next used the filter functionality to eliminate the redundant terms (using the default redundancy cutoff of 0.5) to list the enriched terms. The proteins annotated with each of these terms is given in Extended Data File 2.

### Isolation of microglia by FACS

Microglia were purified as previously described [45]. Centrifuges, buffers, and tools were all prechilled to 4 °C or on ice. 7-week-old APPPS1-21 male and female mice treated either with vehicle or ABX were perfused using ice cold Hank’s balanced salt solution (HBSS) and the brains were quickly dissected and placed on ice. Brains were minced using a scalpel and then Dounce homogenized in ice cold HBSS 15–20 times each with the loose and tight pestles. The cell suspension was then transferred to prechilled 50 mL tubes and passed through a pre-wet (with HBSS) 70 μm cell strainer (Miltenyi Biotech). Cell suspensions were then transferred into a prechilled 15 mL tube and spun down at 300 g for 5 min in a centrifuge set to 4 °C. Stock isotonic Percoll (SIP) was prepared by diluting Percoll in 10 × HBBS (10:1). 30%, 37% and 70% Percoll solutions were prepared by diluting SIP with 1 × HBSS. The cell pellet was resuspended in 4 mL of 37% Percoll and transferred to 15 mL tubes. 4 mL of 70% Percoll was underlaid using a syringe and 30% Percoll was layered on top of 37% Percoll followed by 2 mL of 1 × HBSS. The tubes were centrifuged for 30 min at 500 g with no brake. Myelin and other debris were removed by vacuum suction and ~ 2 mL of 70–37% interphase was transferred into clean 15-mL tube and diluted 3 times with 1X HBBS. The samples were centrifuged for 5 min at 500 g at 4 °C. This was repeated 3 times. The cell pellet was washed with 10 mL of ice cold HBSS and spun again for 5 min at 300 g at 4 °C. All samples were then resuspended in 500 μl of ice cold FACS buffer (0.5% BSA, 1 mM EDTA, in 1 × PBS, Sterile Filtered) containing CD11b (M1/70 PE; Biolegend 101,208), and CD45 (APC Biolegend, 103,116), antibodies from Biolegend at a 1:200 dilution for 30 min on ice. Samples were then washed in 10 mL of ice cold FACS buffer and spun down for 5 min at 300 g and then resuspended in 500 μl of ice cold FACS buffer. Microglia were then sorted on a BD FACS Aria II using the 70-micron nozzle with purity mode into individual wells with a sort speed of approximately 10,000 events per second. Each sample took approximately 5–10 min to sort. The sorted microglial cells were immediately proceeded with isolation of RNA using Trizol reagent. The cDNAs for qPCR using primers specific for *Hexb*, *Calm1*, *Atp8* and *Atp6v0d1* were performed as mentioned previously in the q(RT-PCR) analysis. For expression analysis 2^^ΔΔCT^ method [46] was used wherein the difference between C_T_ values of the gene interest and reference gene (*Gapdh*) is calculated for all the samples and normalized to WT-Veh_M.

### Metabolomic analyses

#### Metabolite extraction from cecal material

Extraction solvent (80% methanol spiked with internal standards and stored at -80 °C) was added to pre-weighed fecal/cecal samples at a ratio of 100 mg of material/mL of extraction solvent in beadruptor tubes (Fisherbrand; 15–340-154). Samples were homogenized at 4 °C on a Bead Mill 24 Homogenizer (Fisher; 15-340-163), set at 1.6 m/s with 6 thirty-second cycles, 5 seconds off per cycle. Samples were then centrifuged at 10 °C, 20,000 x g for 15 min and the supernatant was used for subsequent metabolomic analysis.

#### *A. Short-chain fatty acid analysis*

Short chain fatty acids were derivatized as described by Haak et al. with the following modifications [47]. The metabolite extract (100 μL) was added to 100 μL of 100 mM borate buffer (pH 10) (Thermo Fisher, 28,341), 400 μL of 100 mM pentafluorobenzyl bromide (Millipore Sigma; 90,257) in Acetonitrile (Fisher; A955-4), and 400 μL of n-hexane (Acros Organics; 160,780,010) in a capped mass spec autosampler vial (Microliter; 09–1200). Samples were heated in a thermomixer C (Eppendorf) to 65 °C for 1 h while shaking at 1300 rpm. After cooling to RT, samples were centrifuged at 4 °C, 2000 × g for 5 min, allowing phase separation. The hexanes phase (100 μL) (top layer) was transferred to an autosampler vial containing a glass insert and the vial was sealed. Another 100 μL of the hexanes phase was diluted with 900 μL of n-hexane in an autosampler vial. Concentrated and dilute samples were analyzed using a GC–MS (Agilent 7890A GC system, Agilent 5975C MS detector) operating in negative chemical ionization mode, using a HP-5MSUI column (30 m × 0.25 mm, 0.25 μm; Agilent Technologies 19091S-433UI), methane as the reagent gas (99.999% pure) and 1 μL split injection (1:10 split ratio). Oven ramp parameters: 1 min hold at 60 °C, 25 °C per min up to 300 °C with a 2.5 min hold at 300 °C. Inlet temperature was 280 °C and transfer line was 310 °C. A 10-point calibration curve was prepared with acetate (100 mM), propionate (25 mM), butyrate (12.5 mM), and succinate (50 mM), with 9 subsequent 2 × serial dilutions. Data analysis was performed using MassHunter Quantitative Analysis software (version B.10, Agilent Technologies) and confirmed by comparison to authentic standards. Normalized peak areas were calculated by dividing raw peak areas of targeted analytes by averaged raw peak areas of internal standards.

#### *B. Indole and B vitamin analysis*

Indole-containing metabolites, B-vitamins and other targeted metabolites were analyzed by LCMS/MS. The metabolite extract (400 μL) was added to pre-labeled microcentrifuge tubes. Samples were dried down completely using a Genevac EZ-2 Elite. Samples were resuspended in 100 μL of 50:50 Water: Methanol and added to an Eppendorf thermomixer. C at 4 C, 1000 rpm for 15 min to resuspend analytes. Samples were then centrifuged at 4 °C, 20,000 × g for 15 min to remove insoluble debris. The supernatant (80 μL) was transferred to a fresh, prelabeled MS vial with inserts or 96 deep-well plate (Agilent 5065–4402). Samples were analyzed on an Agilent 1290 infinity II liquid chromatography system coupled to an Agilent 6470 triple quadrupole mass spectrometer, operating in positive mode, equipped with an Agilent Jet Stream Electrospray Ionization source. Each sample (2 μL) was injected into a Acquity UPLC HSS PFP column, 1.8 μm, 2.1 × 100 mm (Waters; 186,005,967) equipped with a Acquity UPLC HSS PFP VanGuard Precolumn, 100., 1.8 μm, 2.1 mm X 5 mm (Waters; 186,005,974) at 45 °C. Mobile phase A was 0.35% formic acid in Water and mobile phase B was 0.35% formic acid in 95:5 Acetonitrile: Water. The flow rate was set to 0.5 mL/min starting at 0% B held constant for 3 min, then linearly increased to 50% over 5 min, then linearly increased to 95% B over 1 min, and held at 100% B for the next 3 min. Mobile phase B was then brought back down to 0% over 0.5 min and held at 0% for re-equilibration for 2.5 min. The QQQ electrospray conditions were set with capillary voltage at 4 kV, nozzle voltage at 500 V, and Dynamic MRM was used with cycle time of 500 ms. Transitions were monitored in positive mode for 46 analytes. An 11-point calibration curve (ranging from 0.88 nM to 909 μM) was prepared for tryptophan, tyrosine, phenylalanine, serotonin, 5-HIAA, melatonin, tryptamine, kynurenine, kynurenic acid, anthranilic acid, and niacin. Data analysis was performed using MassHunter Quant software (version B.10, Agilent Technologies) and confirmed by comparison with authentic standards. Normalized peak areas were calculated by dividing raw peak areas of targeted analytes by averaged raw peak areas of internal standards.

### Illustrations

Schematic in Fig. 9 was created using Biorender.

## Results

### Generation and characterization of ‘APPPS1-21-CD11br’ TRAP transgenic mice

**Fig. 1:**
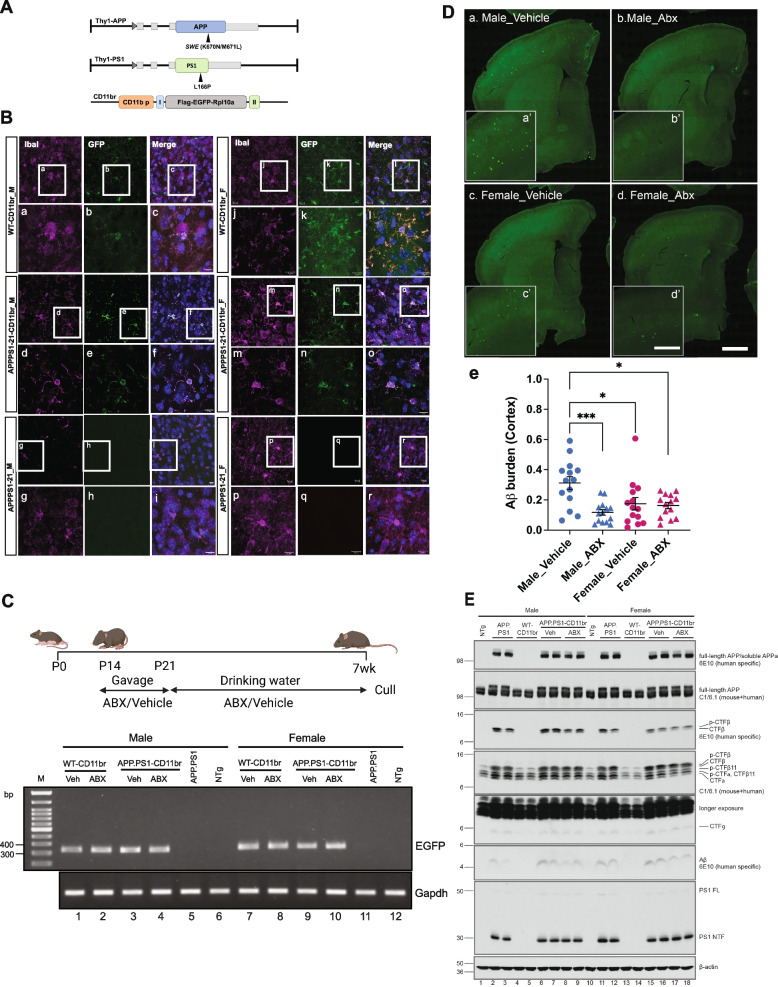
Effect of ABX-treatment on sex-specific cerebral amyloidosis of APPPS1-21-CD11br transgenic mice. **A** Schematic representation of APP/PS1 transgenes and FLAG/EGFP tagged murine Rpl10a under CD11b promoter. I, intervening sequence; II, SV40 polyA. **B** EGFP expression in brain sections of WT-CD11br, APPPS1-21-CD11br and APPPS1-21 male and female mice. GFP immunostaining co-localized with macrophage/microglial marker, Iba I. **C** Schematic to represent the treatment regime. Expression of FLAG-EGFP-Rpl10a transgene from cortical RNAs of WT and APPPS1-21-CD11br mice treated with vehicle or ABX by RT-PCR. An amplicon of 0.3 kb corresponding to EGFP in the transgene was detected on 1% agarose gel. APPPS1-21 and non-transgenic mice were used as control. *Gapdh* was used as a reference gene. **D** Representative images of Aβ plaque burden in the cortex of vehicle and ABX-treated in 7-wk-old APPPS1-21-CD11br mice using anti-Aβ monoclonal antibody, 3D6 (left). Quantification of plaque burden in vehicle and ABX-treated APPPS1-21-CD11br mice using threshold-limited particle analysis of 3D6 positive staining from six sections per case and expressed relative to the total cortical area of each slice (right, *n* = 15). **E** Detection of the expression of human APP and PS1 transgenes in detergent-soluble brain lysates of 7-wk-old male and female APPPS1-21-CD11br mice by Western blot analysis. 6E10, a human APP-specific antibody detected steady-state levels of human full-length APP, soluble APP, -CTF and Aβ peptides. C1/6.1 antibody detected both mouse and human full-length APP and CTFs. The expression of human PS1L166P was confirmed by PS1NT antibody and β-actin was used as loading control. NTG (non-transgenic) and APPPS1-21 served as controls (*n* = 2)

**Fig. 2:**
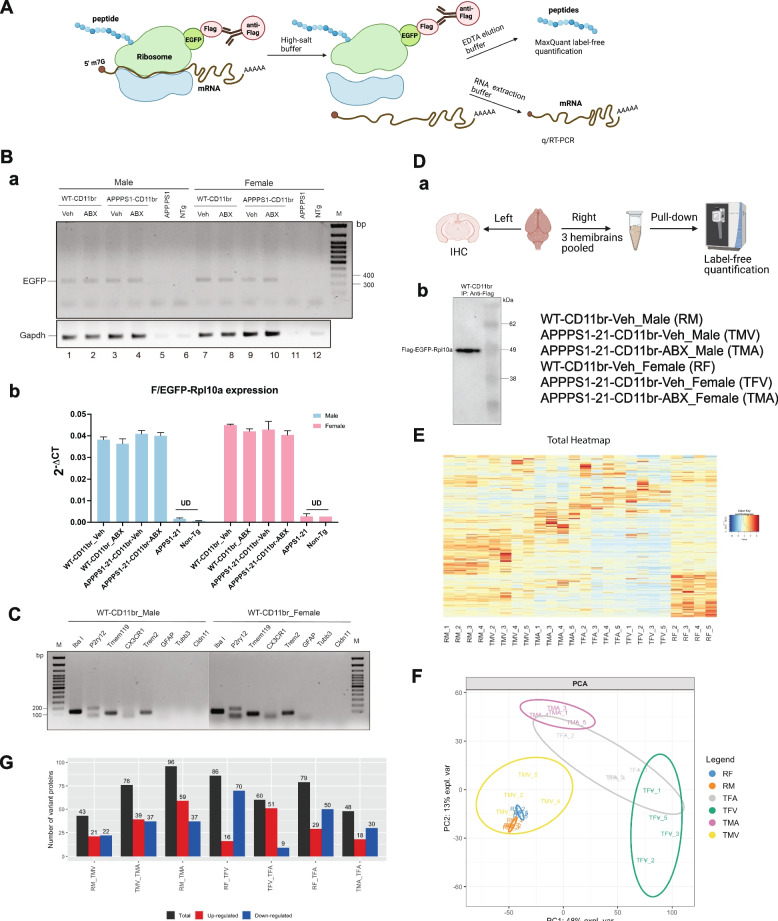
Identification of microglial translatome and LFQ quantification using MaxQuant.**A** Schematic representation of the ribosome affinity purification method from CD11br mice brain cortical tissue. **B** cDNAs synthesized from the purified RNA after immunoprecipitation of cortical lysates from WT and APPPS1-21-CD11br treated with vehicle or ABX was used for RT-PCR analysis. An amplicon corresponding to EGFP in the FLAG/EGFP-Rpl10a transgene mRNA was detected (**a**). No band was detected in APPPS1-21 and non-Tg samples that were used as controls for pull-down assay. Gapdh was used as reference gene. (**b**) qPCR analysis with the same primer set showed that the levels of EGFP were comparable between all the mice groups. 2^Δ ΔCT^ method was used to show the expression of FLAGEGFP-Rpl10a transgene wherein the difference between C_T_ values of the gene interest and reference gene is calculated for all the samples and plotted as a bar graph. The error bar indicates the SD of two technical replicates. UD refers to undetermined. **C** RT-PCR analysis of cDNAs from WT-CD11br male and female mice indicate amplification of microglia/macrophage-specific markers *Iba1*, *P2ry12*, *Tmem119*, *Cx3cr1*and *Trem2*. *Gfap*, *Tubb3*, *Cldn11* were used as markers for astrocytes, neurons, and oligodendrocytes respectively. **D** Six-cohorts of mice including vehicle-treated WT-CD11br male (RM); CD11br female (RF); vehicle-treated APPPS1-21-CD11br male (TMV); vehicle-treated APPPS1-21-CD11br female (TFV); ABX-treated APPPS1-21-CD11br male (TMA); and ABX-treated APPPS1-21-CD11br female (TFA) mice were employed for proteomic analysis of the newly-synthesized peptides in microglia. Animals were sacrificed at 7 weeks of age (total *n* = 15, per group) and 3 half cortices from 3 mice were pooled into each sample based on their amyloid burden for each cohort (*n* = 5). Western blot after immunoprecipitation of cortical lysates from WT-CD11br mice with anti-flag antibody demonstrates the immunobinding of the F/EGFP-Rpl10a transgene to FLAG beads (**b**). **F** Principal component analysis (PCA) plot based on normalized data of the proteins identified from all six groups of the proteomic analysis. **G** Venn diagram representing the number of proteins found in all replicates of each group of male mice (RM, TMV, TMA) and female mice (RF, TFV, TFA). **H** Number of differentially expressed proteins (DEPs) for each comparison, black-total number of significant DEPs (Limma q-value < 0.05 and |z|> 1.96); red and blue indicate upregulated and downregulated proteins respectively

To identify the in vivo cell-type specific protein profiles from CD11b + microglial/macrophage cells and address the physiological basis underlying their phenotypic transition, we crossed APPPS1-21 mice that express FAD-linked APP_swe_ and PS1_L166P_ transgenes driven by the neuron-specific Thy1-promoter [26] to CD11brGFP mice [36] to generate ‘APPPS1-21-CD11br’ mice. The CD11brGFP mice express a transgene encoding FLAG-EGFP-tagged ribosomal protein L10a (Rpl10a) driven by the human microglia/macrophage-specific CD11b promoter (Fig. 1A). The expression of transgenes was confirmed by PCR amplification of genomic DNA from APPPS1-21-C11br mice using primer sets specific for human APP and human PS1 (SI. Figure 1A) and for FLAG/EGFP-Rpl10a (F/EGFP-Rpl10a) (SI. Figure 1B). We first confirmed the expression of F/EGFP-Rpl10a was specific to CD11b + microglia/macrophages, by immunofluorescence of EGFP and CD11b/IBAI markers (SI.Figure 2A). Next, we analyzed the CD11b + cells using double immunofluorescence analysis for CD11b and P2RY12/TMEM119, two known microglia-specific markers in WT-CD11b and APPPS1-21-CD11br mice; Aβ-specific 3D6 antibody was used to stain Aβ plaques in APPPS1-21-CD11br mice (SI. Figure 2B, C). SI. Figure 2D further shows overlap of GFP with P2RY12 in both WT-CD11br and APPPS1-21-CD11br mice. We also performed immunofluorescence analysis for GFP to confirm the expression of F/EGFP-Rpl10a in both male and female WT-CD11br and APPPS1-21-CD11br mice. As shown in Fig. 1B, there is an overlap of GFP with endogenous IBA1-positive macrophage/microglial cells. APPPS1-21 mice were used as controls to demonstrate the absence of GFP fluorescence that would otherwise be associated with the presence of *F/EGFP-Rpl10a* transgene.

### ABX-treatment results in reduced Aβ pathology in male APPPS1-21-CD11br mice

To investigate the effect of gut microbiome on Aβ amyloidosis in APPPS1-21-CD11br mice, we followed our established ABX-treatment regime wherein male and female animals are gavaged with a high dose of ABX cocktail postnatally (P14-P21) followed by low dose ABX (1:50 dilution) in drinking water until they are sacrificed at 7-weeks, an early stage of Aβ deposition [24] (Schematic, Fig. 1C).

To address if ABX-treatment has any effect on the expression of *F/EGFP-Rpl10a* transgene, we isolated total RNAs from cortical lysates of WT, vehicle, or ABX-treated male and female APPPS1-21-CD11br mice and performed RT-PCR using primers specific for. As shown in Fig. 1C, there was no detectable change in the levels of EGFP mRNA after ABX-treatment. Using 3D6, an Aβ -specific monoclonal antibody, we confirmed that at 7-weeks, Aβ deposits are predominantly cortical, as was previously described in APPPS1-21 mice [24, 26] (Fig. 1D). ABX-treated male APPPS1-21-CD11br mice exhibited significant reductions in cortical Aβ burden compared with the vehicle-treated counterparts. On the other hand, vehicle-treated female mice showed lower Aβ levels compared with vehicle-treated male mice at this age and ABX had an insignificant change in Aβ burden in female mice, as was previously reported in female APPPS1-21 mice [24].

To confirm that the ABX-mediated decrease in the Aβ levels in male APPPS1-21-CD11br mice is not due to differences in APP levels, we performed Western blot analysis of detergent-soluble extracts using human APP-specific 6E10 antibody or C1/6.1 antibodies that recognize both human and endogenous mouse APP. We show that in both male and female mice, the expression of full-length human APP transgene is unchanged after ABX-treatment, whereas the level of Aβ in brain cortex is reduced in ABX-treated male mice following treatment (Fig. 1E). Interestingly, ABX-treated female mice appeared to exhibit a slight decrease in Aβ levels compared with vehicle-treated animals even though the levels of plaque staining by IHC using the 3D6 antibody did not show significant changes. As the detergent soluble fraction contains a fraction of deposited Aβ monomeric and “soluble” Aβ oligomers, it is conceivable that the ABX lowering effects on Aβ in females is a reflection of selective alterations in monomeric and oligomeric species that are not deposited. Further studies will be necessary to validate this hypothesis.

### Translational profiling of microglia/macrophage reveals sex-specific expression profiles in the cerebral cortex of APPPS1-21-CD11br mice

**Fig. 3:**
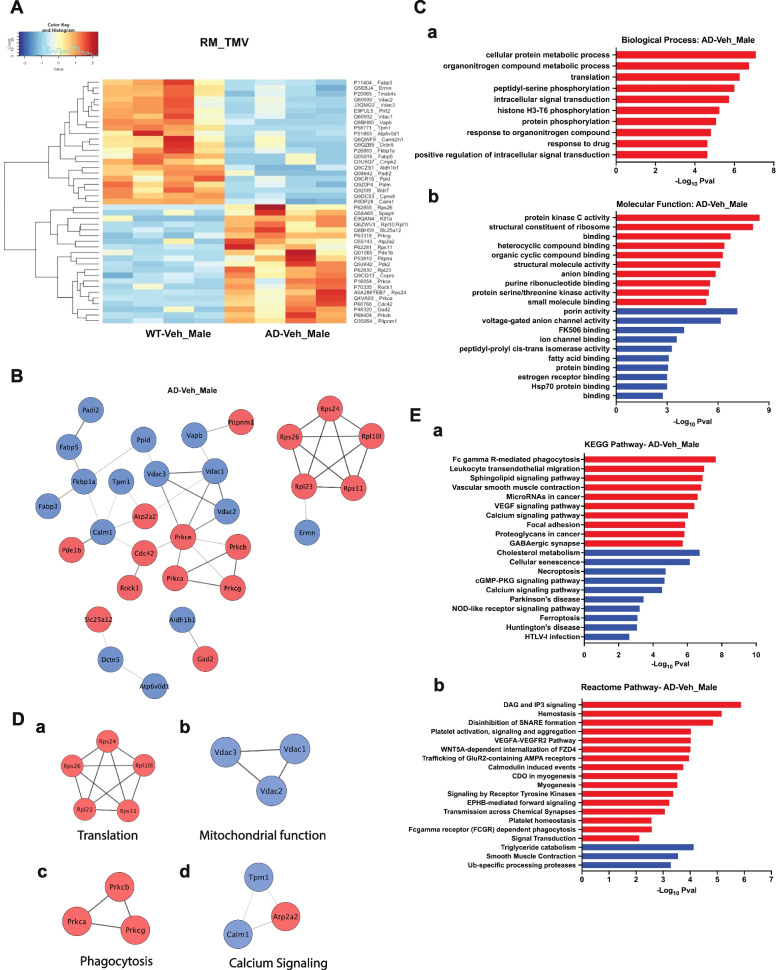
Functional analysis of DEPs in male APPPS1-21-CD11br mice (TMV). **A** Proteomic heatmap profiles with hierarchical clustering to represent the intensity values of the significant DEPs identified by pairwise comparative analysis between APPPS1-21-CD11br (TMV) and WT-CD11br (RM) male mice. **B** Protein–protein interaction (PPI) networks were constructed using STRING with DEPs in APPPS1-21-CD11br male mice. Nodes represent proteins, edges represent interactions between two proteins. Proteins containing single node and not involved in the network are not included. **C** Significant terms from GO Biological Process (**a**) and Molecular Function (**b**) associated with Functional enrichment analysis of upregulated (red) and downregulated (blue) DEPs that form PPI network. **D** Significant protein clusters (**a**-**d**) identified by MCODE analysis of the PPI network of proteins.** E** KEGG (**a**) and Reactome (**b**) pathway enrichment analysis using STRING to identify the significant pathways of DEPs from APPPS1-21-CD11br male mice (FDR > 0.05) that form PPI network

Translation of mRNAs into proteins involved in innate immune responses is a tightly regulated process mediated by several post-transcriptional mechanisms [48]. To assess the molecular signatures of microglial phenotypes in response to ABX-treatment observed from our previous studies, we employed our APPPS1-21-CD11br mice with a modified TRAP approach that allow assessment of the microglial/macrophage transcriptome and proteome (Fig. 2A). To first confirm that our assay is selective for CD11b + microglial/macrophage ribosomes and that CD11b promoter activity is not altered in a different genetic background or upon ABX-treatment, cortical tissue homogenates were immunoprecipitated using anti-FLAG agarose beads and polysome complexes were used for mRNA purification. Following cDNA synthesis using dT_18_, RT-PCR analysis was performed using primers specific for EGFP in order to show that the steady-state expression of mRNA encoding F/EGFP-RPL10A protein is at similar levels in both male and female APPPS1-21-CD11br mice. APPPS1-21 and non-transgenic (non-Tg) mice were used as controls to establish specificity of the pull-down assay (Fig. 2B, a). qPCR analysis confirmed that the expression level of FLAG-tagged ribosomes and the pull-down efficiency is comparable between different genotypes, treatment, and sex (Fig. 2B, b). Next, to confirm that the pull-down is specific for CD11b + polysomes, we used the cDNAs from WT-CD11br male and female mice for RT-PCR analysis using primers specific for microglia/macrophage markers *IbaI*, *P2ry12*, *Tmem119*, *Cx3cr1* and *Trem2*. As controls, we employed primers specific for *Gfap*, *Tubb3*, *Cldn11* as markers for astrocytes, neurons, and oligodendrocytes, respectively (Fig. 2C). As shown in SI. Figure 3A (a, b), *Tmem119* and *P2ry12* were also detected in cDNAs prepared from immunoprecipitated polysomes from WT and APPPS1-21 CD11br male and female mice treated with ABX or vehicle. As both parenchymal microglia and CNS-associated macrophages (CAMs) such as perivascular macrophages, are the tissue-resident myeloid cells in CNS that also express CD11b, we performed RT-PCR analysis with primers specific for *Hexb*, a bonafide marker for microglia and *CD163*, that is expressed at high levels in CAMs. In SI. Figure 3B panel a, we show that the RNA from the pull-down of CD11b + cells is enriched for microglia as indicated with high abundance of *Hexb* mRNA, as compared to CAMs that are characterized by high expression of *CD163*. This is further confirmed by relatively lower ΔCt value of *Hexb* compared to *CD163* by qPCR analysis (SI. Figure. 3B, panel b).

To assess the proteomic signatures of microglial phenotypes associated with their activation in our APPPS1-21-CD11br model, we employed our CD11b microglia/macrophage reporter mice and performed proteomic analysis of newly-synthesized peptides (Fig. 2D, a). Six groups of mice were included in the analysis: vehicle-treated WT-CD11br male (RM); WT-CD11br female (RF); vehicle-treated APPPS1-21-CD11br male (TMV); vehicle-treated APPPS1-21-CD11br female (TFV); ABX-treated APPPS1-21-CD11br male (TMA); and ABX-treated APPPS1-21-CD11br female (TFA) mice. Animals were sacrificed at 7 weeks of age (*n* = 15, per group). In view of the low number of microglia in the brain [49], and our use of the low-expressing F/EGFP-RPL10a transgenic mice line [36], we chose pooled cortices from 3 hemi-brains with similar Aβ burdens for each sample (Please see additional note in the methods section). Immunoprecipitation of cortical homogenates was performed using anti-FLAG agarose beads and the polysome complexes were used for peptide extraction for LC-MSMS followed by proteomic analysis with label-free quantification (LFQ) of the newly-synthesized peptides, as previously described [36]. Western blot analysis of immunoprecipitated polysomes from WT-CD11br confirmed the presence of the FLAG-tagged EGFP-RPL10a polypeptide, thus confirming the binding of F/EGFP-RPL10a by anti-Flag beads (Fig. 2D, b).

**Fig. 4:**
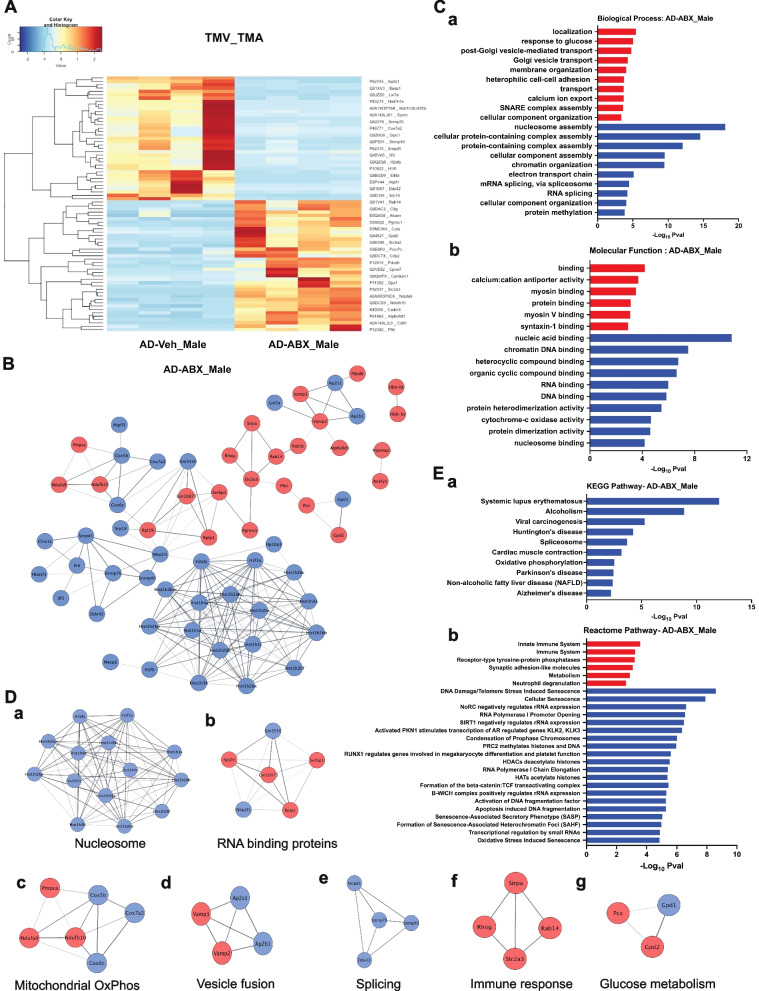
Functional analysis of DEPs in ABX-treated male APPPS1-21- CD11br mice (TMA). **A** Proteomic heatmap profiles with hierarchical clustering to represent the scaled-intensity values of the significant DEPs identified by pair-wise comparative analysis between ABX-treated (TMA) and vehicle-treated male APPPS1-21-CD11br (TMV) mice in all biological replicates. **B** Protein–protein interaction (PPI) networks were constructed using STRING with DEPs in ABX-treated male APPPS1-21-CD11br mice. Nodes represent proteins, edges represent interactions between two proteins. Proteins containing single node and not involved in the network are not included. **C** Significant terms from GO Biological Process (**a**) and Molecular Function (**b**) associated with Functional enrichment analysis of upregulated (red) and downregulated (blue) DEPs that form PPI network.** D** Significant protein clusters (**a**-**g**) identified by MCODE analysis of the PPI network of DEPs in ABX-treated male AD mice.** E** KEGG (**a**) and Reactome (**b**) pathway enrichment analysis was done using STRING to identify the significant pathways of microglial proteins from ABX-treated APPPS1-21-CD11br male mice (FDR > 0.05) involved in PPI network

Heatmap with unsupervised hierarchical clustering based on the protein abundances shows an overall reproducibility as well as individual heterogeneity of protein expression profiles within each sample across all groups of mice (Fig. 2E). Principal component analysis (PCA) plot, based on normalized data of the whole data set revealed that the proteomic profiles in male mice groups (RM, TMV, TMA) were distinct whereas female mice exhibited significant overlap between the vehicle (TFV) versus ABX-treatment (TFA) groups, thus indicating similar protein profiles (Fig. 2F). To account for sex-differences in gene expression, we compared within male and female mice groups to address the effect of transgene/Aβ expression and ABX-treatment on the microglial proteome: (i) Vehicle-treated AD transgenic with WT mice (RM_TMV & RF_TFV) (ii) ABX vs vehicle-treated AD transgenic (TMV_TMA & TFV_TFA) and (iii) ABX-treated transgenic mice with WT mice (RM_TMA & RF_TFA). In total, we identified 2,182 proteins and of these, 1560 proteins were quantifiable i.e., proteins with at least 80% of observed intensities in each replicate in one of the two groups of comparison. To add confidence, proteins with at least 2 peptides were quantified on each pair-wise comparison (SI. Figure 4A). Venn diagrams of the quantified proteins from all 4 samples of each group that are shared between male and female mice are shown in SI. Figure 4B. Statistical analyses revealed the differentially expressed proteins (DEPs) for each of the group comparison between male and female mice (FDR < 0.05, students t-test) (Fig. 2G). List of all the DEPs is presented in Extended Data File 1.

### Differential expression analysis reveals proteins associated with mitochondrial dysfunction and calcium signaling in male APPPS1-21-CD11br mice

**Fig. 5:**
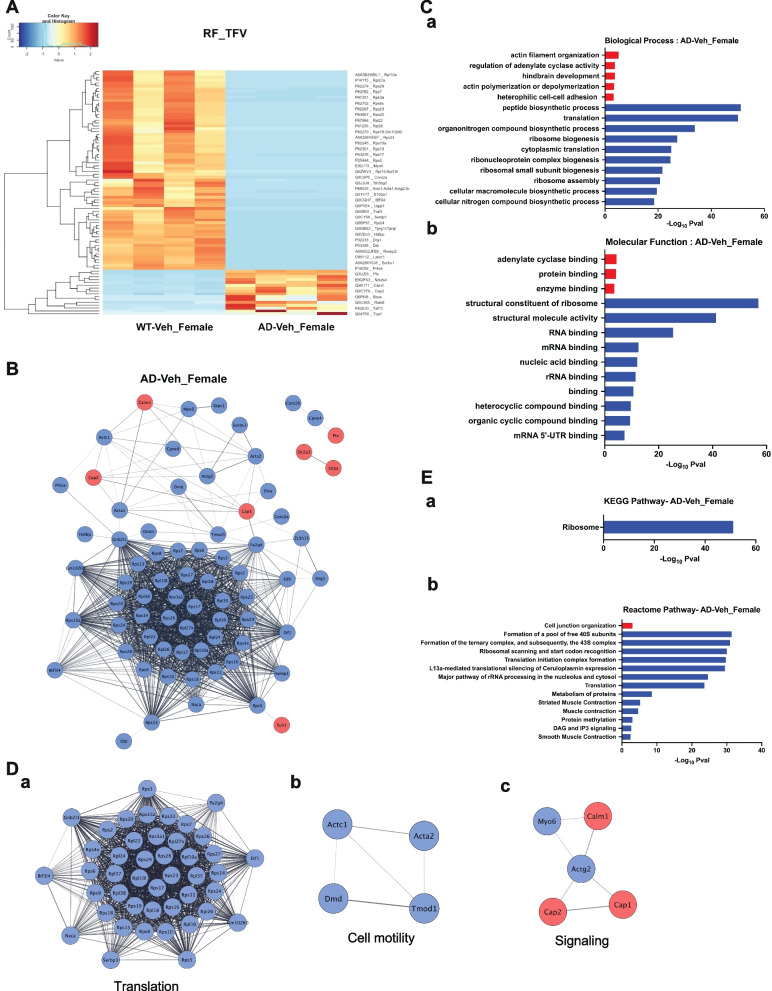
Functional analysis of DEPs in female APPPS1-21-CD11br mice (TFV). **A** Proteomic heatmap profiles with hierarchical clustering to represent the intensity values of the significant DEPs identified by pair-wise comparative analysis between APPPS1-21-CD11br (TFV) and WT-CD11br (RF) female mice in all biological replicates. **B** Protein–protein interaction (PPI) networks were constructed using STRING with DEPs in APPPS1-21-CD11br female mice. Nodes represent proteins, edges represent interactions between two proteins. Proteins containing single node and not involved in the network are not included. **C** Significant terms from GO Biological Process (**a**) and Molecular Function (**b**) associated with Functional enrichment analysis of upregulated (red) and downregulated (blue) DEPs that form PPI network. **D** Significant protein clusters (**a-c**) identified by MCODE analysis of the PPI network of DEPs in female AD mice.** E** KEGG (**a**) and Reactome (**b**) pathway enrichment analysis was done using STRING to identify the significant pathways of microglial proteins from APPPS1-21-CD11br female mice (FDR > 0.05) involved in PPI network

Statistical analysis of the quantified proteins between male APPPS1-21-CD11br (TMV) and WT-CD11br (RM) mice revealed that expression of the FAD-linked APP_swe_ and PS1_L166P_ transgenes significantly altered the expression of 86 microglial/macrophage proteins. Heatmaps with hierarchical clustering were constructed with statistically significant 21 upregulated and 22 downregulated DEPs (Fig. 3A). The top 10 DEPs are listed in SI. Table 1. To identify known and potential protein–protein interactions (PPI) relevant to changes in protein expression due to microglial activation, we used STRING [43] database that quantitatively integrates protein data from multiple sources. The PPI networks for upregulated and downregulated DEPs for male mice included 44 nodes connecting with 47 edges (Fig. 3B) and their enriched GO terms for Biological Process (BP) and Molecular Function (MF) are presented in Fig. 3C, a-b. An enrichment map for visualization of GO terms and %terms per group generated by ClueGo [41] and CluePedia [42] tools of Cytoscape is given in SI. Figure 5A and B. Next, we analyzed cluster networks using MCODE, a plugin that identifies highly interconnected regions or clusters in a protein interaction network [44]. Our analysis revealed 4 MCODE clusters (MC) (Fig. 3D). MC1 (Rps24, Rps26, Rpl23, Rps11, Rpl10l) correlated to GO categories linked to translational machinery components. MC2 (Vdac1, Vdac2, Vdac3) was linked to mitochondrial function. MC3 (Prkca, Prkcb, Prkcg) linked to protein phosphorylation and phagocytosis. Atp2a2, Calm1, Tpm1 involved in calcium signaling formed MC4.

Aβ peptides, in addition to direct neurotoxic effects, can bind to pattern recognition receptors on microglia and induce signaling transduction pathways to mediate an inflammatory response and also leads to phagocytosis [50]. Our data identifies ribosomal proteins-components of the translational machinery as upregulated, and previous studies on proteomic analysis of frontal cortex of AD patients showed GO categories linked to the ribosome [51]. On the other hand, Vdacs, known to regulate the metabolic and energetic functions of the cell, including calcium homeostasis and oxidative stress [52] are downregulated. While Atp2a2, an ATPase pump present on ER that regulates calcium ion homeostasis [53] is upregulated, Calm1, a sensor to modulate ER contacts with Atp2a2 [54] and Tpm1 which regulates microglial pro-inflammatory phenotype downstream of TREM2 via PKA signaling [55], are downregulated.

### FcγR-mediated phagocytic pathway is enriched in male APPPS1-21-CD11br

To understand the biological role of the DEPs in male APPPS1-21-CD11br mice, functional enrichment analysis was performed using KEGG and Reactome pathways (Fig. 3E). Pathway analysis revealed that upregulated proteins were significantly enriched in FcγR-mediated phagocytosis (Cdc42, Prkca/b/e/g), and DAG and IP3 signaling pathways (Pde1b, Prkce/g), and downregulated proteins in triglyceride catabolism (Fabp3/5, Vapb), and calcium signaling (Calm1) pathways. All the significant pathways are shown in Extended Data File 2.

Microglia express several receptors that function to recognize, internalize, and clear Aβ and for activation [14]. Fc receptors (FcR), the ITAM-associated receptor family members modulate innate immune responses by phagocytosis or by releasing cytokines. FcγR-mediated phagocytosis involves two important events (i) remodeling of the cytoskeleton and (ii) activation of oxidative bursts [56]. Binding of ligands, including Aβ, to FcR on microglial plasma membrane leads to receptor clustering [57]. This trigger signaling events including tyrosine phosphorylation by Protein kinase C (PKC), activation of Phospholipase C to cleave phosphatidylinositol phosphates (PIP_2_) into diacylglycerol (DAG) and inositol triphosphate (IP_3_), for actin remodeling in the formation of phagosome.

Taken together, these results indicate that microglial proteins in male APPPS1-21-CD11br mice related to mitochondrial dysfunction, and fatty-acid metabolism are associated with Aβ pathology and Aβ -induced cellular toxicity. The upregulation of proteins involved in FcγR-mediated phagocytosis indicates that microglial activation likely leads to internalization and subsequent degradation of Aβ peptides.

### Differential expression analysis shows modulation of immune responses in ABX-treated male APPPS1-21-CD11br mice

Statistical analysis of the quantified proteins between ABX (TMA) and vehicle-treated male (TMV) APPPS1-21-CD11br mice revealed that ABX-treatment significantly altered the expression of 76 proteins (Fig. 4). Heatmaps with hierarchical clustering were constructed with 39 upregulated and 37 downregulated DEPs (Fig. 4A). The top 10 DEPs are shown in SI. Table 2.

Among the most upregulated DEPs, SIRPα, a microglial surface receptor interacts with its ligand CD47, the ‘*Don’t Eat me*’ signal, constitutes cell–cell communication via the CD47-SIRPα signaling axis [58] and regulates receptor-mediated phagocytosis [59]. Gpd2 shuttles with glycerol phosphate to integrate microbial stimulation with glucose oxidation to balance the induction and suppression of inflammatory response by macrophages [60]. The most downregulated proteins included histone core proteins (H2afy, H3f3a) and linker proteins (Hist1h1e/Hist1h1c) indicating decondensation of chromatin, presumably for driving transcription of genes encoding immune modulators.

**Fig. 6:**
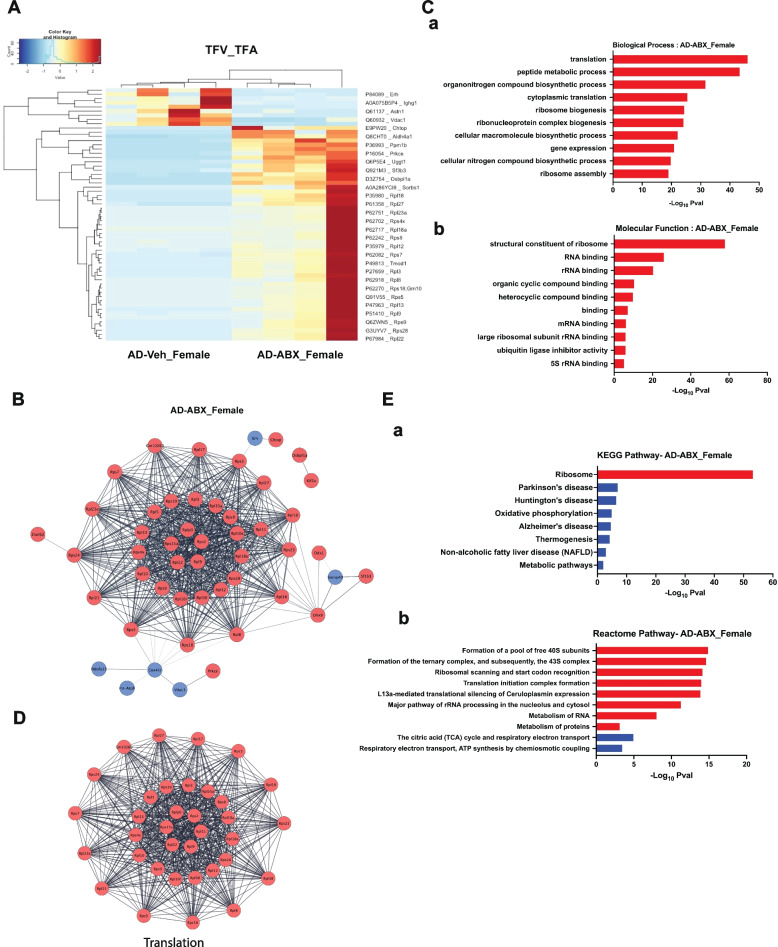
Functional analysis of DEPs in ABX-treated female APPPS1-21-CD11br mice (TFA). **A** Proteomic heatmap profiles with hierarchical clustering to represent the scaled-intensity values of the significant DEPs identified by pair-wise comparative analysis between ABX-treated (TFA) and vehicle-treated APPPS1-21-CD11br (TFV) female mice in all biological replicates. **B** Protein–protein interaction (PPI) networks were constructed using STRING with DEPs identified from ABX-treated female APPPS1-21-CD11br mice. Nodes represent proteins, edges represent interactions between two proteins. Proteins containing single node and not involved in the network are not included. **C** Significant terms from GO Biological Process (**a**) and Molecular Function (**b**) associated with Functional enrichment analysis of upregulated (red) and downregulated (blue) DEPs that form PPI network. **D** Significant protein clusters identified by MCODE analysis of the PPI network of DEPs in ABX-treated female AD mice. **E** KEGG (**a**) and Reactome (**b**) pathway enrichment analysis was done using STRING to identify the significant pathways of microglial proteins from ABX-treated female APPPS1-21-CD11br mice (FDR > 0.05) involved in PPI network

PPI networks for upregulated and downregulated DEPs included 91 nodes connecting 194 edges (Fig. 4B). The GO terms, biological process (BP), and molecular functions (MF), that are associated with these proteins are shown in Fig. 4C. A larger-scale perspective of the biological functions associated with all the DEPs identified in ABX-treated male APPPS1-21-CD11br mice were visualized in an enrichment map (SI. Fig. 6). MCODE analysis revealed 7 clusters of protein interactions. MC1 showed 14 nodes of histone proteins that correlated to GO categories associated with nucleosome assembly. MC2 (Serbp1, Rpl29, Rplp1, Gm3550, Gm10078, Nhp2l1) and MC3 (Pmpca, Ndufa9, Ndufb10, Cox5b, Cox7a2, Cox6c) are linked to localization and electron transport chain. MC4 (Vamp2, Vamp3, Ap2s1, Ap2b1) and MC5 (Snrpd1, Snrnp70, Snrnp40, Ddx42) are linked to vesicle assembly and mRNA splicing. Sirpa, Rhog, Slc2a3, Rab14 formed MC6 that are linked to immune response and MC7 (Pcx, Gpd1, Gpd2) correlated to GO categories linked to glucose metabolism (Fig. 4D, a-g).

### ABX-treated male APPPS1-21-CD11br mice show coordination of metabolic and epigenetic pathways

Pathway enrichment analysis of ABX-treated male APPPS1-21-CD11br mice (Fig. 4E) revealed that upregulated proteins were enriched in innate immunity (Atp6v0d1, Pfkl, Rab14, Slc2a3, C8g, Cd81, Pgrmc1, Rhog) and metabolic (Pfkl, Rab14, Slc2a3, Cpne7, Ndufb10, Gpx1, Ndufa9, Pcx, Gpd2, Slc25a12) pathways. Downregulated proteins were associated with OXPHOS (Cox6c, Cox7a2, Cox5b), cellular senescence and DNA methylation (Hist1h2bb, Hist1h1e, Hist1h1c, Hist1h2bm, H3f3a) and splicing (Snrpd1, Snrnp40, Snrnp70, Ddx42) (Fig. 4E).

Proteins from ABX-treated male APPPS1-21-CD11br mice also showed upregulation of the glycolytic pathway, receptors for glucose uptake, and transport. Accumulating evidence suggests that different metabolic pathways are required for programming of microglial polarization [61]. Activation of microglia induces anerobic glycolysis that involves an increase in glucose uptake, driving production of lactate from pyruvate. In parallel, pentose phosphate pathway generates NADPH for NADPH oxidase required for production of Ribose-5-phosphate, a precursor for synthesis of nucleotides [62]. Downregulated pathways were associated with senescence, Senescence-Associated-Secretory Phenotype (SASP) and formation of Senescence-Associated-Heterochromatin foci (SAHF). Oxidative stress and DNA damage induce cellular senescence and chronically activated microglia are known to adopt SASP that triggers an inflammatory cascade [63]. The switching to more efficient bioenergetic substrates decreases oxidative stress by ROS, resulting in decreased cell and DNA damage.

Taken together, the microglial proteins in ABX-treated male APPPS1-21-CD11br mice revealed that modulation of immune responses by microglial polarization results from the regulation of several protein networks. These include changes in metabolism that impact cellular reprogramming by production of essential metabolites and supporting the specific metabolic demand.

### Differential expression analysis reveals downregulation of ribosomal proteins in female APPPS1-21-CD11br mice

Statistical analysis of the quantified proteins between female APPPS1-21-CD11br (TFV) and female WT-CD11br (RF) mice revealed 172 DEPs that met the significance threshold. Heatmaps with hierarchical clusters were constructed using 16 upregulated and 70 downregulated DEPs (Fig. 5A). The top 10 DEPs are shown in SI. Table 1. Among the most upregulated proteins, Top1, DNA Topoisomerase I, is shown to be highly expressed during microglial activation and in neuroinflammatory state [64]. Ndufs4, NADH Dehydrogenase (Ubiquinone) Fe-S protein 4 (Ndufs4) is one of the subunits of mitochondrial complex I [65], and Sirpa mediates immune responses [58]. On the other hand, ribosomal proteins were among the most downregulated polypeptides.

**Fig. 7:**
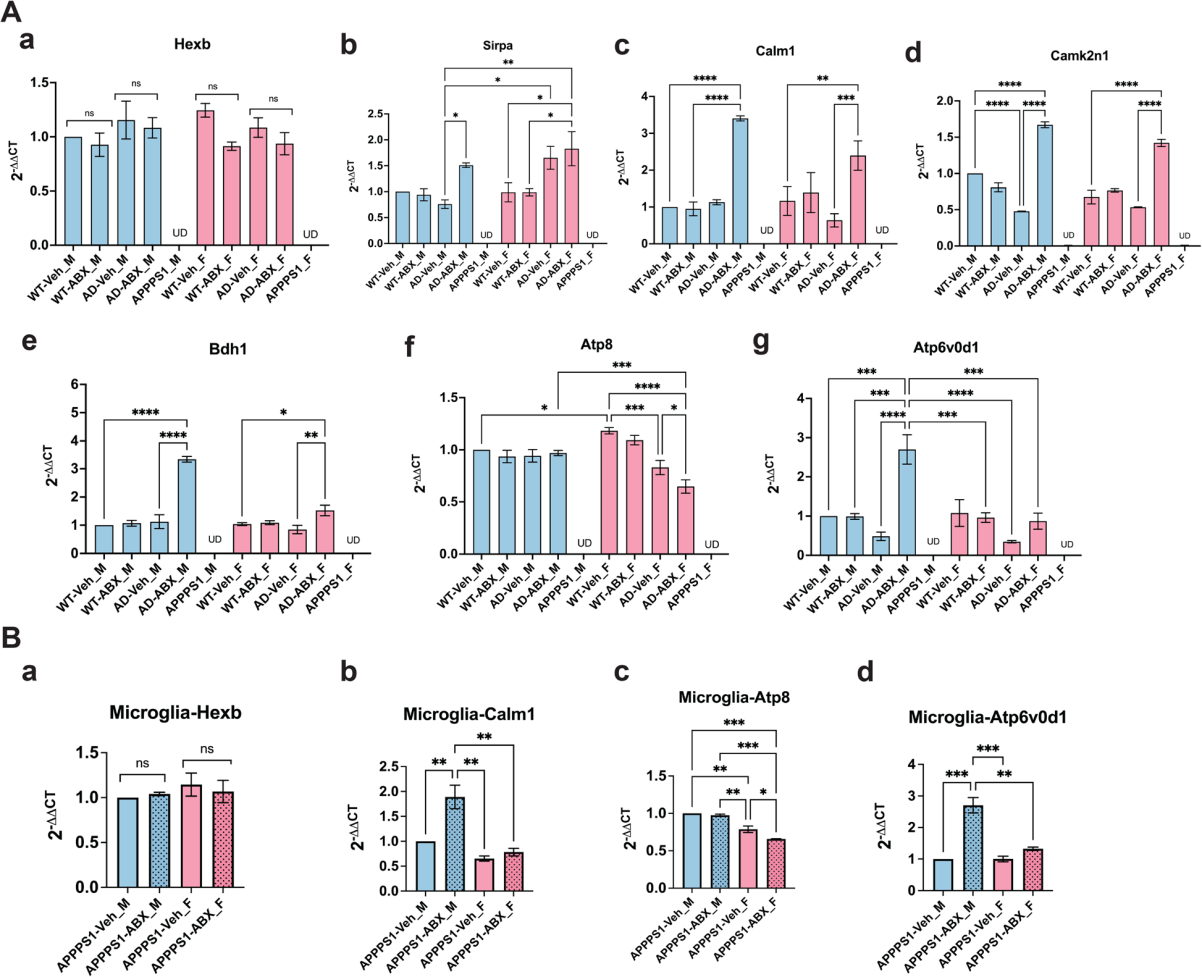
qPCR analysis to validate significant microglial transcripts identified by translatome profiling. **A** cDNAs synthesized from the purified RNA after immunoprecipitation of cortical lysates from vehicle or ABX-treated WT-CD11br and vehicle or ABX-treated APPPS1-21-CD11br male and female mice (*n* = 3 per group) were used for qPCR analysis. APPPS1-21 male and female mice were used as controls for pull-down assay. *Gapdh* was used as reference gene. The relative expression levels of microglia transcripts *Hexb* (**a**, bonafide microglia marker), *Sirpa* (**b**, immune response), *Calm1*, *Camk2n1 *(**c-d**, Calcium Signaling), *Bdh1*(**e**, Ketone metabolism), *Atp8* (**f,** Mitochondrial ATP synthase) and *Atp6v0d1*(**g**, lysosomal v-ATPase) are determined using qPCR by the ΔΔCT method and plotted as a bar graph normalized to the expression of WT-vehicle treated male mice. The error bar indicates the SD of two technical replicates. UD refers to undetermined. **B** *Hexb, Calm1, Atp8* and *Atp6v0d1* transcripts were chosen for further validation using cDNA prepared from RNA purified from FACS-isolated microglia. APPPS1-21 male and female mice treated with vehicle or ABX were sacrificed at 7-weeks. 3-hemicortices from each group were pooled. Following Percoll-density centrifugation, Flow-cytometry was used to isolate CD11b^hi^ + CD45 ^lo^ cells (enriched in microglia). The cells were immediately used for RNA isolation using Trizol reagent. The relative expression of *Hexb* (**a**), *Calm1*(**b**), *Atp8* (**c**) and *Atp6v0d1* (**d**) was determined by ΔΔCT method and plotted as a bar graph normalized to the expression of vehicle-treated male AD (APPPS1-21) mice. The error bar represents the SD of two experimental replicates. Statistical significance of the effect of ABX-treatment and sex on expression of the transcripts were indicated as *p < 0.05; **p < 0.01; ***p < 0.001; ****p < 0.0001; ns-not significant, based on Tukey’s Multiple comparison test. ns indicates no significance

Next, the PPI networks for upregulated and downregulated DEPs for ABX-treated female APPPS1-21-CD11br mice showed 90 nodes connecting 1012 edges (Fig. 5B). The GO terms (BP, MF) associated with these proteins are shown in Fig. 5C. An enrichment map to visualize the GO terms associated with all the DEPs in female AD mice is given in SI. Figure 7. MCODE analysis revealed 3 clusters with MC1 showed the largest module with 44 nodes including the top5 downregulated Rps11, Rps5, Rps9, Rpl38, Pa2g4, Rpl27a linking 929 edges corelated to GO categories linked to peptide biosynthetic process and ribosome biogenesis. MC2 (Actc1, Acta2, Dmd, Tmod1) and MC3 (Cap1, Cap2, Calm1, Actg2, Myo6) are linked to actin organization and adenylate cyclase activity (Fig. 5D, a-c).

### Functional enrichment analysis reveals pathway associated with nucleolar stress response in female APPPS1-21-CD11br mice

Pathway analysis revealed that upregulated proteins were associated with cell-junction organization (Flna, Cadm3) (Fig. 5E). Downregulated proteins that were largely ribosomal proteins were enriched in pathways associated with translation including formation of 40S ribosome, translation initiation complex, rRNA processing and metabolism of proteins. Interestingly, DAG and IP3 signaling pathways (Prkce, Calm1) in ABX-treated female APPPS1-21-CD11br mice were downregulated as opposed to male counterparts where these pathways are upregulated. In AD, nucleoli, the ribosome factories of cells formed around transcription and maturation of rRNAs are reported to be dysfunctional [66]. As primary mediators of cellular stress response they optimize energy consumption by inhibiting rRNA gene transcription and ribosome assembly as a response to protein aggregation due to hampered proteasome function. Aβ42 oligomers increase oxidative stress and a gradual accumulation of nucleolar stress in a concentration dependent manner, resulting in altered production of ribosomes, protein translation, rRNA, rRNA oxidation and hypermethylation [67].

Together, our analyses show that in female APPPS1-21-CD11br mice, there is an enrichment of microglial/macrophage proteins involved in translation and RNA surveillance pathways that are instigated by the nucleolar stress response.

### Antibiotic-treated female APPPS1-21-CD11br mice show restored expression of ribosomal proteins

**Fig. 8:**
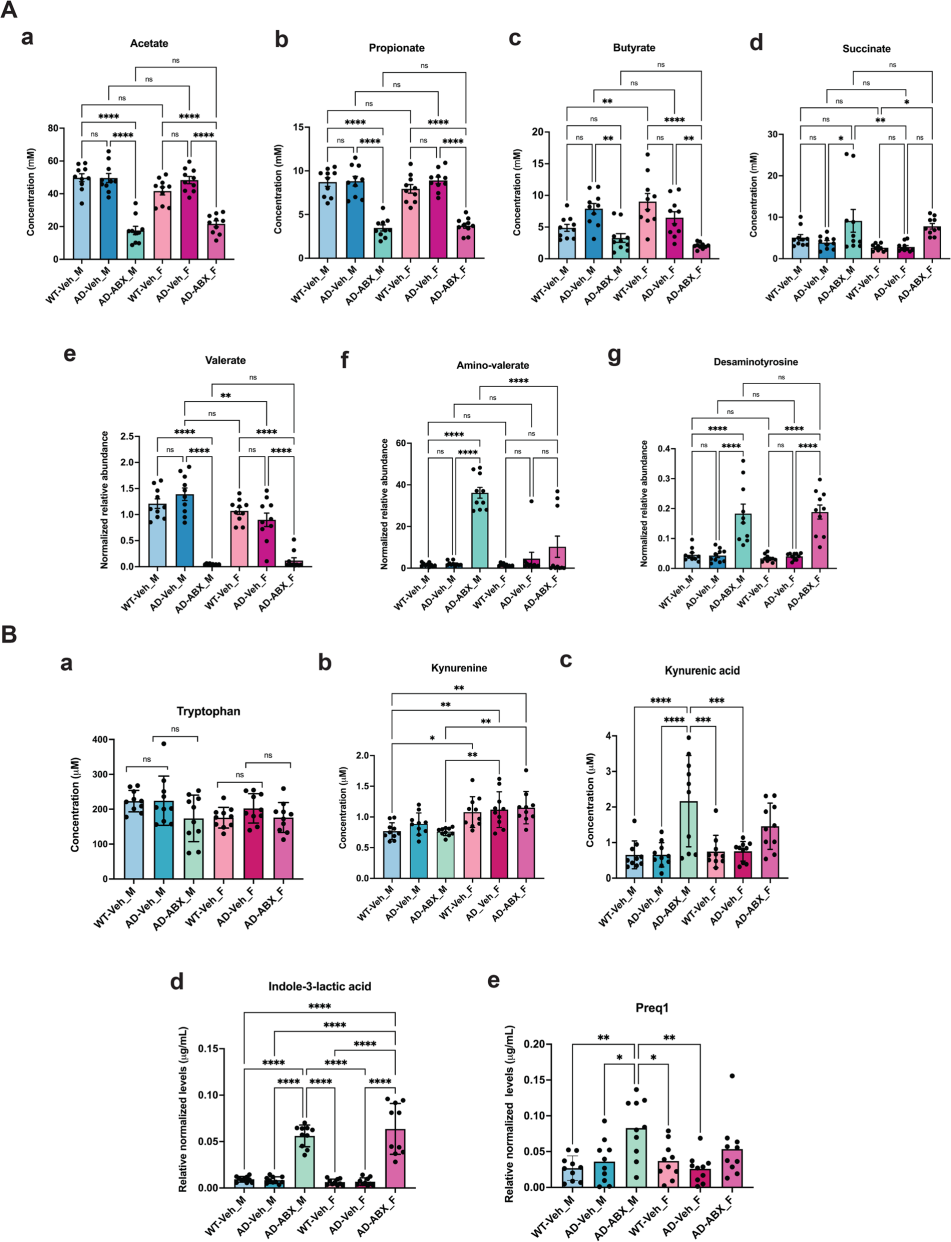
Effect of ABX-treatment on cecal metabolite abundances in APPPS1-21-CD11br mice at 7-weeks. Targeted GC–MS analysis of cecal metabolites in male and female APPPS1-21-CD11br mice treated with ABX or vehicle (*n* = 10 per group) tested by one-way ANOVA analysis. Statistical significance of the effect of treatment and sex on (**A**) SCFA metabolites and (**B**) Tryptophan metabolite levels were indicated as (adjusted *p*-val: p) *p < 0.05; **p < 0.01; ***p < 0.001; ****p < 0.0001; ns-not significant, based on Tukey’s Multiple comparison test. The concentrations of most abundant SCFAs acetate, propionate, butyrate, and succinate (**A**, panels **a**-**d**) and normalized relative abundances of less abundant metabolites valerate, amino valerate and Desamino-tyrosine (**A**, panels **e**–**g**) in male and female WT, AD mice treated with vehicle or ABX mice are shown. The concentrations of significantly altered tryptophan metabolites Kynurenine, Kynurenic acid, Indole-3-lactic acid, and PreQ1 are shown in **B**, panels **a**-**c** respectively

Statistical analysis of the quantified DEPs between TFA and TFV revealed that ABX treatment in APPPS1-21-CD11br female mice resulted in 51 upregulated and 9 downregulated proteins. Figure 6A shows heatmap with hierarchical clustering of statistically significant DEPs. The top 10 upregulated are listed in SI. Table 2. Among the top 3 upregulated are ribosomal proteins Rps9, Rps5 and Rps17. Importantly, microglial secreted protein UDP-glucose: glycoprotein glucosyl transferase (Uggt1) that forms a hub in the chaperone network of the endoplasmic reticulum (ER), and acts as a protein quality control sensor, is upregulated [68]. Mt-atp8 and Ighg1 which interact with FcγR for phagocytosis were the most downregulated DEPs. Notably, the mitochondrial ATP-synthase, Mt-Atp8, involved in both synthesis and hydrolysis of ATP during OXPHOS was identified as an oxidatively-damaged protein in patients with early stages of AD [69]. Next, the PPI networks for the significant DEPs included 60 nodes connecting with 623 edges (Fig. 6B). The GO terms (BP, MF) associated with these proteins are shown in Fig. 6C. An enrichment map to visualize the biological functions associated with all the DEPs in ABX-treated female APPPS1-21-CD11br mice is shown in SI. Figure 8. MCODE analysis revealed one significant cluster, MC1 with 34 nodes corresponding to ribosomal proteins linking 560 edges. Rpl13, Rps5, Rps9, Rpl3, Rplp0, Rpl35a are identified as hub proteins each with ~ 110 interactions (Fig. 6D).

### Microglial/macrophage translatome reveals impaired mitochondrial function in ABX-treated female APPPS1-21-CD11br mice

Pathway enrichment analysis revealed upregulated proteins were enriched in ribosome, rRNA processing in nucleolus, and cytosol and metabolism of proteins (Uggt1, Ank2, Rps9, Rps18, Rps3, Rps4x, Rps23, Rps7, Rps2, Rps28, Rps15a) (Fig. 6E). Metabolically active cells require high levels of ribosomal RNAs that are essential for maintenance of cellular protein content. Ribosomal transcription and nucleolar integrity are sensitive to environmental changes such as nutrient deprivation, temperature fluctuations and stress signals like oxidative damage [67, 70]. The nucleolus, a hub for temporal storage for unfolded proteins and a protein quality control center, serves to restore the epigenomic landscape after proteotoxic stress is lifted [71]. Downregulated pathways were associated with proteins in the citric acid (TCA) cycle, electron transport chain, and ATP synthesis by chemiosmotic coupling (Mt-Atp8, Ndufa13, Vdac1). Dysfunctional mitochondrial respiration is known to inhibit specific responses associated with alternative activation of microglia [72].

Taken together, the microglial/macrophage proteins in ABX-treated female APPPS1-21-CD11br mice revealed upregulation of nucleolar mediated ribosomal RNA and cytoplasmic proteins processing pathways, albeit with compromised bioenergetics due to decrease in mitochondrial OXPHOS.

### Validation of mRNA levels of key microglial DEPs by qPCR

As we profiled the actively translating peptides that are enriched for microglia, we chose to validate the key DEPs identified by our proteomic analysis by qPCR of mRNAs that were being actively translated. We prepared RNA purified from immunoprecipitated polysomes from cortical homogenates of WT and APPPS1-21 CD11br male and APPPS1-21 CD11br female mice treated with vehicle or ABX, prepared cDNAs from each RNA sample, then analyzed the relative expression of specific RNAs by the ΔΔCt method. RNA from APPPS1-21 mice were used as a control for the pull-down assay (Fig. 7A, panels a-g). First, using qPCR analysis we confirmed that there is no significant change in the expression of the microglia-specific marker *Hexb* due to treatment or sex (Fig. 7A, panel a). We chose specific primers to analyze the expression of (i) *Sirpa*, involved in microglial activation and in neuroinflammation via SIRPα-CD47 signaling [58], (ii) Calm1 involved in Calcium signal transduction pathways in innate immune responses by regulating enzymes, and ion-channels [73], and (iii) *Camk2n1*, an endogenous inhibitor of CaMKII, a crucial regulator of inflammatory responses that modulates the production of NF-κB, IL-10, IL-2, and IL-4 [74]. *Sirpa* was relatively higher in ABX-treated AD male mice compared to vehicle-treated males and in females, this mRNA showed higher expression in both vehicle and ABX-treated AD mice compared to WT (Fig, 7A, panel b). The levels of *Calm1* and *Camk2n1* were higher in both ABX-treated male and female APPPS1-21-CD11br mice compared with their vehicle-treated counterparts (Fig, 7A, panels c, d). We then analyzed mRNA encoding mitochondrial D- β -hydroxybutyrate dehydrogenase (*Bdh1*), a key enzyme of ketone metabolism that was identified as an upregulated DEP in ABX-treated male APPPS1-21-CD11br mice. *Bdh1* mRNA was nearly threefold higher in ABX-treated male APPPS1-21-CD11br mice while it was only a moderate increase was observed in ABX-treated female APPPS1-21-CD11br mice compared with vehicle-treated animals (Fig. 7A, panel e). Next, we analyzed *Mt-Atp8*, a F-type proton-translocating ATPase (F-ATPase) involved in mitochondrial OXPHOS that we identified as a DEP downregulated in ABX-treated female APPPS1-21-CD11br mice. While the male mice did not show significant changes in expression upon ABX treatment, we observe there was a decrease in the expression in female APPPS1-21-CD11br mice compared with WT animals, which was further lowered in ABX-treated female APPPS1-21-CD11br mice (Fig. 7A, panel f). Lastly, we analyzed the expression of *Atp6v0d1*, the ATPase H + Transporting V0 Subunit D1**,** a component of lysosomal v-ATPase identified as a DEP whose expression was restored upon ABX-treatment in male APPPS1-21-CD11br mice. As shown in Fig. 7A, panel g we observed the expression of *Atp6v0d1* was ~ twofold higher in ABX-treated male APPPS1-21-CD11br mice compared with vehicle-treated male APPPS1-21-CD11br mice, while the female APPPS1-21-CD11br mice showed no significant changes between animals treated with ABX or vehicle.

**Fig. 9:**
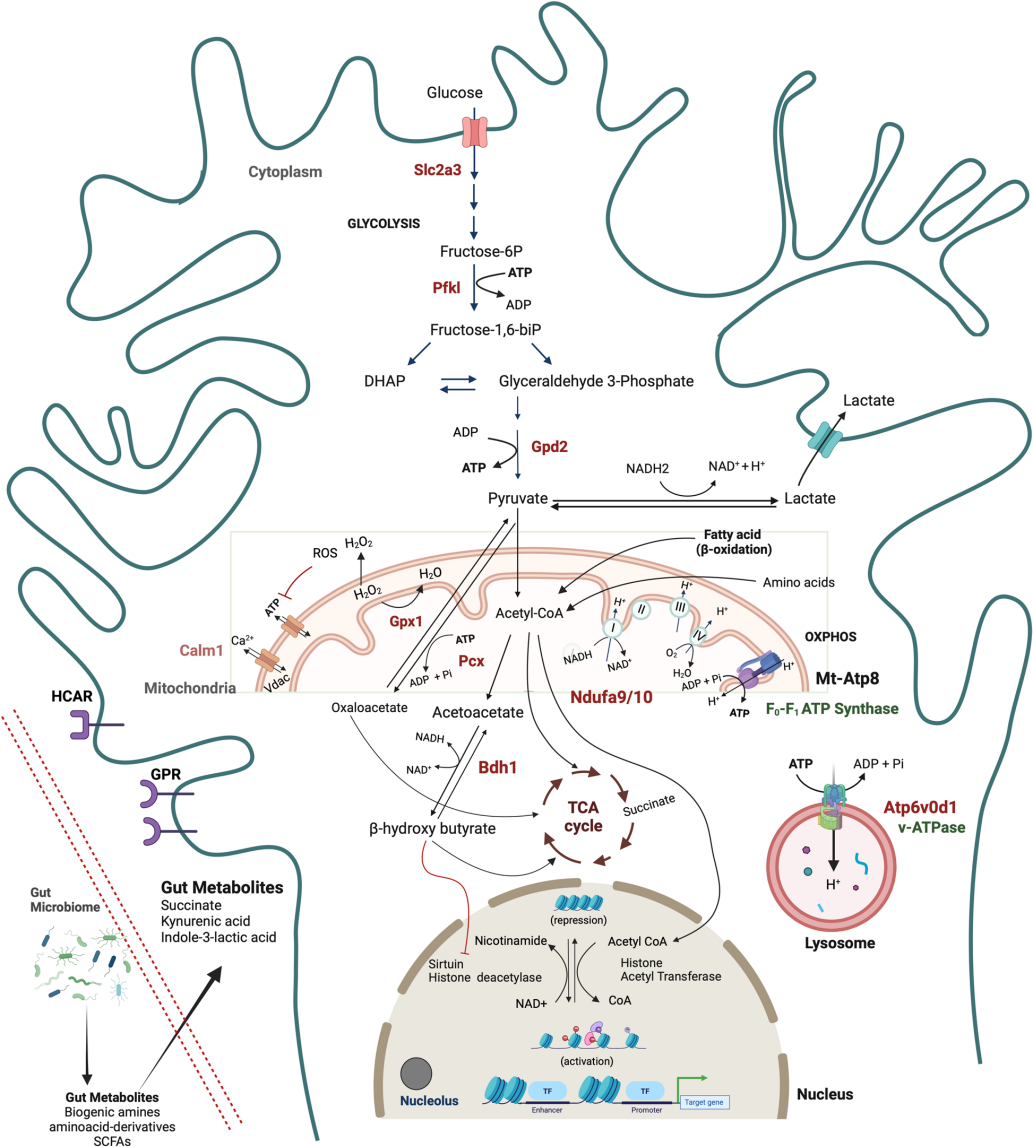
ABX-treatment induced changes in gut-metabolites provides metabolic support for microglial neuroprotective phenotype in male AD mice. Schematic to show metabolic and epigenetic coordination of microglial translatome in ABX-treated APPPS1-21-CD11br male mice**.** The upregulated DEPs identified from the proteomic analysis are represented in red (bold). Microglial polarization states and immune functions are regulated by their metabolic adaptation. ABX-treatment showed increase in Slc2a3 (glucose transporter), Pfkl (glucose metabolism), Gpd2 (Calcium-responsive, increases glucose oxidation to fuel the production of acetyl co-A); Pcx (catalyzes formation of oxaloacetate required for biosynthetic pathways); Bdh1 (interconversion of acetoacetate and β-hydroxybutyrate, the two major ketone bodies produced during fatty acid metabolism); Ndufa9, Ndufb10 (NADH: ubiquinone oxidoreductase, Complex I, Electron transport Chain to synthesize ATP); Gpx1 (antioxidant activity). This metabolic shift can support production of stable intermediates to maintain the oxidative status and energetics of the cell. Acetyl-CoA that serves as universal substrate for the acetylation of histones by Histone acetyl transferases (HAT) results in open chromatin structure for positive transcriptional regulation. NAD^+^ functions as a cofactor for Sirtuin (SIRTs) histone deacetylase (HDAC) enzymes leading to heterochromatin formation [92]. Metabolites derived from gut bacteria (ligands for Aryl hydrocarbon receptor (AHR) & G protein coupled receptors (GPR)) including SCFAs like succinate, amino-acid derivatives like desaminotyrosine and Tryptophan metabolites like Kynurenic acid and Indole-3-lactic acid can improve the cellular energy state required for microglial immune modulation. The rescue in the expression of Atp6v0d1 (lysosomal v-ATPase component) in ABX-treated male AD mice indicates that this metabolic flexibility is crucial for mitochondrial and lysosomal crosstalk to facilitate Aβ clearance by lysosomal acidification

To further confirm the above findings are specific for microglial cells, we isolated total RNA from microglia purified by Percoll density centrifugation and FACS-purification (CD11b^hi^-CD45^lo^), prepared cDNAs and performed qPCR analysis of mRNAs encoding *Hexb*, *Calm1*, *Atp8* and *Atp6v0d1* [45]. Here, microglia were purified from cortical homogenates of 7-week-old male and female APPPS1-21-CD11br mice treated with ABX or vehicle (SI. Figure 9). qPCR analysis confirmed that there was no significant change in the expression of *Hexb* due to treatment or sex (Fig. 7B, panel a). Consistent with our earlier data (Fig. 7A, panel c), ABX-treatment increased the expression of *Calm1* in male mice only (Fig. 7B, panel b). Furthermore, and as we observed above (Fig. 7A, panel f), while there were no significant changes in the levels of *Atp8* mRNA between ABX- and vehicle-treated APPPS1-21 CD11br male mice, we observed a decrease in ABX-treated APPPS1-21 CD11br female mice compared to the vehicle-treated animals (Fig. 7B, panel c). Lastly, and as we observed above (Fig. 7A, panel g), we show ~ 2.5-fold increase in the expression of mRNA encoding *Atp6v0d1* in ABX-treated APPPS1-21 male mice compared with the vehicle-treated counterparts while no significant changes in expression of *Atp6v0d1* mRNA were observed in female APPPS1-21 mice that were treated with ABX or vehicle (Fig. 7B, panel d). Taken together, our qPCR analysis validates the expression of microglial Calm1, Mt-Atp8 and Atp6v0d1 that were identified as significant DEPs from our translational profiling. We posit that these polypeptides might be critical in mediating sex-specific immune responses by modulating microglial metabolism.

### ABX-treatment alters immunomodulatory cecal metabolites in a sex-dependent manner

Recent studies suggest that microbiome modulates microglial activation through production of microbial gut metabolites including short-chain fatty acids (SCFAs) [20, 75]. The metabolic output of gut microflora is determined by its composition, and many other factors including diet, activity, and stress. The state of the host’s immunity can also impact upon gut metabolite production. Consistent with our previous reports, cecal weight measurements showed that ABX-treated male and female APPPS1-21-CD11br mice exhibited larger ceca at the time of the sacrifice compared with vehicle-treated counterparts (SI. Figure 10A). To determine the changes in gut metabolites resulting from ABX-mediated changes, we performed GC and LC–MS on cecal contents to quantify various metabolites.

Differences in the levels of metabolites from fatty acids, bile acid and amino-acid derivatives are shown in a heatmap of normalized values and are expressed as relative fold-change (SI. Figure 10B, b-d). While the levels of major SCFAs such as acetate, propionate, and butyrate and a minor SCFA valerate were low in ABX-treated male and female APPPS1-21-CD11br mice compared with their vehicle-treated treated counterparts, the levels of succinate were higher specifically in ABX-treated APPPS1-21-CD11br male mice compared with vehicle-treated male animals (Fig. 8A, panels a-e). The levels of 5-aminovalerate, a bacterial metabolite of lysine catabolism was higher only in ABX-treated male APPPS1-21-CD11br mice compared with vehicle treated male APPPS1-21-CD11br mice (Fig. 8A, panel f). Desamino tyrosine (DAT), a microbial metabolite of tyrosine degradation, is at higher abundance in both ABX-treated male and female APPPS1-21-CD11br mice compared with the vehicle treated counterparts (Fig. 8A, panel g). Next, we analyzed the effect of ABX on metabolites of tryptophan catabolism (Fig. 8B, panels a-e). While there were no significant changes in the levels of tryptophan and kynurenine based on treatment or sex, the level of kynurenic acid (KYNA) was specifically higher in ABX-treated APPPS1-21-CD11br male mice compared with vehicle-treated male counterparts. was significantly higher in ABX-treated male and female AD mice compared to their vehicle-treated counterparts. PreQ1, a metabolic intermediate in queuosine pathway, known to be synthesized by gut bacteria, was at higher levels only in ABX-treated male APPPS1-21-CD11br mice. Taken together, our metabolomic analyses indicate that ABX-treatment resulted in changes in specific gut metabolites that might be immunomodulatory and influence metabolic performance based on sex.

## Discussion

Emerging studies continue to expand on the scope of influence of the gut microbiome on several neurological diseases, including Alzheimer’s and Parkinson’s disease. Microglia, the brain-resident macrophages serve as the first active immune barrier in the CNS and acquire distinct phenotypes upon exposure to intrinsic and extrinsic cues in their environment [12, 76]. The spatio-temporal expression profiles of transcriptome and translatome are not linear and show incongruous expression [77]. Therefore, proteomic profile of microglia is crucial to accurately assess biological functions. Here, we investigated the translatome of cortical microglia/macrophage using TRAP of AD ‘APPPS1-21-CD11br’ mice and offer several novel insights into their sex-specific phenotypes and functions associated with their activation. Consistent with our previous report [24], ABX-mediated changes in gut microbiome resulted in decreased cerebral amyloidosis in APPPS1-21-CD11br specifically in male mice (Fig. 1D).

First, our proteomic analysis revealed Aβ peptide-induced toxicity in male APPPS1-21-CD11br mice resulting in mitochondrial dysfunction and dysregulation of calcium signaling (Vdac1, Vdac2, Vdac3, Calm1) (Fig. 3 and SI. Figure 5). Recognition of oligomeric or fibrillar Aβ as host-derived danger-associated molecular patterns (DAMPs) by microglia causes DAMP-induced ion-fluxes, mitochondrial reactive ROS production or lysosomal destabilization and trigger activation of the inflammasome [78]. We show that the male AD mice with upregulated proteins (Cdc42, Prkca, Prkcb, Prkce, Prkcg) are associated with FcγR-mediated phagocytosis [56] (Fig. 3 and SI.Figure 5). At 7 weeks of age, female APPPS1-21-CD11br mice have lower levels of Aβ plaques compared with their male counterparts though there are no differences in the expression of full-length APP and PS1 genes (Fig. 1D). Several studies show that female sex-hormones are neuroprotective in early ages and their decline with age drives disease progression [79]. We show that female AD mice with upregulated proteins (Cap1, Cap2, Flna, Sirpa) are associated with actin filament organization and Slc2a3, a passive facilitated glucose transporter, indicating microglial activation (Fig. 5 and SI. Figure 7). Intriguingly, we also identified a large number of proteins associated with rRNA processing in the nucleolus and ribosomal proteins in the cytoplasm that are highly downregulated. The signal transduction pathways involved in the expression of inflammatory responses and microglial phenotype transition in response to Aβ based on sex still remain largely unclear and further investigations are needed to understand the Aβ-induced oxidative stress in females.

Second, ABX-treatment in male APPPS1-21-CD11br mice upregulated microglial proteins in the glycolytic pathway (Pfkl, Pcx, Gpd2), different ion channels and receptors (Slc2a3, Atp6v0d1, Slc25a12, Slc8a2, Sirpa, Traf3), and mitochondrial NADH dehydrogenases (Ndufa 9/10) (Fig. 4D and SI. Figure 6). This is accompanied by downregulation of senescence pathways and senescent microglia show a reduced capacity for phagocytosis possibly due to its association with less efficiency in energy production [80]. ABX-treated male AD mice showed decreased inflammation and senescence likely due to upregulation of Gpx1-mediated scavenging of ROS [81]. ABX-treatment of female APPPS1-21-CD11br mice showed a notable restoration in the expression of ribosomal proteins in the cytoplasm and nucleolar proteins involved in rRNA processing (Fig. 6 and SI. Figure 8). Because the production of ribosomes is a major metabolic activity, translational arrest that we observe due to Aβ -induced toxicity might provide a window of opportunity for nucleolus-mediated recovery of epigenetic regulators and restored ribosome biogenesis for microglial immune responses [71]. Further, ABX-treated female APPPS1-21-CD11br mice showed decrease in mitochondrial OXPHOS suggesting metabolic dysfunction.

A central component of the microglial/macrophage phenotypic transition involves the reprogramming of metabolic networks that involve innate immune responses [62, 82, 83]. To validate the key relevant DEPs identified from our TRAP assay, we analyzed the expression of the corresponding mRNAs from pulldown of CD11b + macrophage/microglia polysomes and from FACS purified microglia by qPCR (Fig. 7A and B). Supporting our sex-specific metabolic shift identified from our proteomic analysis, ABX-treated male APPPS1-21-CD11br mice showed threefold increase in Bdh1 while female mice showed a modest increase compared with vehicle treated counterparts (Fig. 7A, panel e). Bdh1 catalyzes NAD+ /NADH coupled interconversion of acetoacetate (AcAc) and β-hydroxybutyrate (βOHB), the two main ketone bodies; βOHB also elevated during fasting and ketogenic diet is used as an alternate fuel for the TCA cycle [84]. It appears that ketone metabolism by microglia and the neuroprotective effects of βOHB against microglia activation has a significant role in AD [85]. Further studies are needed to elucidate the mechanistic insights into how this metabolism modulates microglial activity and function.

Third, we show that the levels of Mt-Atp8 mRNA in ABX-treated female are lower compared with their vehicle-treated counterparts while there was no significant difference in the male groups (Fig. 8A, panel f and B, panel c). Mt-Atp8, provides most cellular energy by coupling proton (H^+^) transport derived from oxidative metabolism with ATP synthesis from ADP and inorganic phosphate [86, 87]. Proteomic studies have identified Atp8 as a target for oxidative damage in patients with early stages of AD as well as in metabolic disorders [69].

Fourth, while there were no differences in the expression levels of microglial Atp6v0d1 in female groups treated with vehicle versus ABX, ABX-treated male APPPS1-21-CD11br mice showed higher expression levels compared with vehicle-treated animals (Fig. 8A, panel g and B, panel d). Atp6v0d1, hydrolyzes ATP to provide the energy to acidify the lysosomal lumen and mediate amyloid clearance through phagocytosis [88, 89]. A recent large-scale proteomic study of AD brains highlighted the significance of energy metabolism and mitochondrial function associated with glial activation [90].

Fifth, it is well-established that microglial phagocytosis requires dynamic reorganization of the cytoskeleton that requires high energy. To increase ATP production, a metabolic switch from OXPHOS to anerobic glycolysis in activated microglia, especially from cells surrounding Aβ plaques was reported in AD pathology [61, 62]. However, sustained activation of glycolytic metabolism leads to low ATP production and lactate accumulation resulting in impaired phagocytic clearance. Further, ATP not only supports energy storage but is a vital molecule that regulates microglial function via purinergic signaling [91]. Based on this evidence, it is tempting to speculate that ABX-mediated changes in male APPPS1-21-CD11br supports the metabolic shift via Bdh1-mediated ketone metabolism for alternate activation of a neuroprotective phenotype (Fig. 9). Acidification of microglial lysosomes via v-ATPase mediates the clearance of Aβ post-internalization via FcγR-mediated phagocytosis. However, disruption of energy metabolism due to mitochondrial OXPHOS dysfunction and persistent inflammation in female APPPS1-21-CD11br mice indicates the ongoing metabolic stress that is likely to result in impaired lysosomal function and phagocytic clearance of Aβ.

Finally, intestinal bacteria possess the capacity to metabolize dietary compounds and produce a range of metabolites that can cross the blood–brain-barrier (BBB) and influence CNS function [93]. Microbial products including short-chain fatty acids (SCFAs), biogenic amines, and amino acid derived metabolites can mediate immune response in the host [94]. Therefore, we leveraged targeted metabolomics and investigated the changes in cecal metabolites mediated by ABX-treatment (SI. Figure 10). In doing so, we identified that ABX-treatment increased the levels of the SCFA succinate and tryptophan metabolite kynurenic acid (KYNA) only in ABX-treated male mice (Fig. 8A). Succinate supplementation has been shown to improve oxidative metabolism and cellular energy in mixed glial cells with mitochondrial dysfunction [95] and the kynurenine pathway is known to lead to the production of NAD + that is critical in generating cellular energy and is a key regulator of the neuroimmune system [96].

Interestingly, desaminotyrosine (DAT), a metabolite produced by a subset of intestinal microbes was shown to enhance antiviral IFN responses in mice [97] and Indole-3-lactic acid (ILA), a tryptophan metabolite associated with commensal Bifidobacterium bacteria that decreases intestinal inflammation [98] are increased in both ABX-treated male and female APPPS1-21-CD11br mice. Collectively, our data suggest that ABX-induced alterations in microbiome modulates gut metabolites that can influence neuroimmune metabolism to support the energy requirements that are substantially increased during microglial activation based on sex (Fig. 9). The molecular mechanisms by which the microbial metabolites can influence microglial function warrants further investigation.

Emerging studies suggest loss of metabolic reprogramming results in microglial dysfunction in Alzheimer’s disease [99, 100]. While proinflammatory microglia utilizes glycolysis to promote cytokine synthesis, immunomodulatory microglia increase fatty-acid metabolism that drive anti-inflammatory responses [101]. However, very little is known about the metabolic determinants in microglia and the mechanisms they adopt to different metabolic states in response to various stimuli. Furthermore, there are limited studies investigating the transcriptome as well as the proteome to account for post-transcriptional regulation of gene expression. Our studies using the TRAP methodology provides the first evidence that gut metabolites can support macrophage/microglial metabolic flexibity based on sex in Aβ cerebral amyloidosis models. The complexity of metabolism coupled with the energetics of immune cells requires further research in order to design novel therapeutics for neurodegeneration. Finally, we would like to highlight that the experimental approaches used to investigate microglial translatomes with either the TRAP assay (CD11bGFP) [36] or the RiboTag assay (Cx3cr1^Cre^) [35] that are intended to enrich for microglia, are not unique in targeting these cells. Future studies that employ mice models wherein a microglia-specific promoter, such as the Hexb promoter, drives expression of a tagged ribosomal protein will be critical for assessment of microglial-specific translatome.

## Conclusions

Our studies that identify actively synthesized polypeptides offer the first insights into macrophage/microglial activation in cerebral Aβ amyloidosis models in a sex-specific manner. In summary, we observe that ABX-mediated perturbations of the gut microbiome can induce metabolic and epigenetic microglial reprogramming for regulating immune responses and amyloid clearance based on sex. Finally, we acknowledge that while we have made correlations between ABX-treatment and changes in the microglial/macrophage translatome, there are significant lacunae in understanding the role of bacterial metabolites that modulate microglial function.

It is widely accepted that microglial responses to extracellular Aβ deposition and/or “soluble” Aβ oligomers have a crucial role in promoting neuroinflammation. Sustained microglial functions require an intracellular metabolic pathway switch that depends on the nutrient availability as a source to meet its energetic demand. Changes in microglial activation in response to ABX-induced alterations reinforce the view that intricate signaling by gut microbiome and resulting metabolites mediate a sex-specific epigenetic program that drives immune effector functions. Reprogramming of microglial metabolism could be a promising strategy for maintaining or restoring the physiological functions of microglia in a manner that might halt the progression of AD.

### Supplementary Information

**Additional file 1: SI. Figure 1.** Confirmation of APP/PS1 and FLAG-EGFP-Rpl10a transgenes in APPPS1-21-CD11br mice. A. Genomic DNA from APPPS1-21-CD11br mice was used for PCR amplification using primer set specific for APP, PS1 transgenes. APPPS1-21 mice serve as positive control. B. Schematic to show the binding sites of various primer sets to confirm the presence of FLAG-EGFP-Rpl10a transgene and the amplicon sizes. 1% Agarose gel to show the amplified products. **SI. Figure 2.** Characterization of CD11b+ cells from CD11br TRAP mice. Representative immunofluorescent images showing co-staining of CD11b with GFP and Iba I from brain sections of WT-CD11br and APPPS1-21-CD11br transgenic mice (A), microglia specific P2ry12 (magenta) and Tmem119 (red) markers colocalized with CD11b+ (green) macrophage/microglia cells from brain sections of 7-wks-old WT-CD11br and APPPS1-21-CD11br male mice (B), microglia specific P2ry12 (red) colocalized with CD11b+ (green) and 3D6 (magenta) stained for amyloid plaques in APPPS1-21-CD11br mice (C). (D) GFP (green) immunostaining colocalized with microglia marker P2ry12 (magenta) in brain sections of WT-CD11br, APPPS1-21-CD11br mice and APPPS1-21 that doesn’t show the expression of GFP was used as a control. The top panel of 2B-D shows low magnification and the selected microglia cells indicated in the box are shown at higher in the bottom panel. **SI. Figure 3.** Microglia specific transcripts from pull-down of cortical lysates of CD11br mice 7-week mice. Cortical lysates were used for immunoprecipitation of CD11b+ microglia/macrophage ribosomes from WT and APPPS1-21-CD11br male and female mice treated with vehicle or ABX and then for mRNA purification. Primer sets for Tmem119 (A) and P2ry12 (B) were used for RT-PCR analysis to confirm the pull-down of RNA from CD11b+ cells were abundant in microglia. Cortical lysates from APPPS1-21 and non-Tg mice were used as controls to indicate the specificity of pull-down. To account for the pulldown of CD11b expressing macrophages, following the synthesis of cDNA, primers specific for Hexb, a bonafide microglia marker and CD163, a selective marker of perivascular macrophages were used for RT-PCR and the amplicons were resolved on 1% Agarose gel (C, a). The ΔCt (Ct [Gene]-Ct [Gapdh]) values from qPCR analysis using the same primer sets to indicate the pulldown of CD11b+ cells were enriched for microglia (C, b). **SI. Figure 4.** A. Number of protein quantifications for each group comparison identified by LFQ. B. Venn diagram representing the number of proteins found in all replicates of each group of male mice (RM, TMV, TMA) and female mice (RF, TFV, TFA). **SI. Figure 5.** Gene Ontology and functional enrichment analysis of DEPs in vehicle-treated male AD (APPPS1-21-CD11br) mice using ClueGo and CluePedia plugins of Cytoscape. GO Terms (Biological Process, Cellular component, Molecular function, Immune pathway) associated with the identified proteins. The most significant parent or child term per functional group (kappa score ≥ 0.4) is shown in the functional grouped network as a group title (a). Peptides associated with the same term are represented by a node. Node color represents the class that they belong. Mixed coloring means that the specific node belongs to multiple classes. Upregulated proteins are shown in red and downregulated are shown in blue. Edges show the association of the peptide with the terms. The thickness of the edge reflects the association significance. (b) Pie chart to indicate specific GO terms (% terms per group). **SI. Figure 6.** Gene Ontology and functional enrichment analysis of DEPs in ABX-treated male AD (APPPS1-21-CD11br) mice using ClueGo and CluePedia plugins of Cytoscape. GO Terms (Biological Process, Cellular component, Molecular function, Immune pathway) associated with the identified proteins. The most significant parent or child term per functional group (kappa score ≥ 0.4) is shown in the functional grouped network as a group title (a). Peptides associated with the same term are represented by a node. Node color represents the class that they belong. Mixed coloring means that the specific node belongs to multiple classes. Upregulated proteins are shown in red and downregulated are shown in blue. Edges show the association of the peptide with the terms. The thickness of the edge reflects the association significance. (b) Pie chart to indicate specific GO terms (% terms per group). **SI. Figure 7.** Gene Ontology and Functional enrichment analysis of DEPs in vehicle-treated female AD (APPPS1-21-CD11br) mice using ClueGo and CluePedia plugins of Cytoscape. GO Terms (Biological Process, Cellular component, Molecular function, Immune pathway) associated with the identified proteins. The most significant parent or child term per functional group (kappa score ≥ 0.4) is shown in the functional grouped network as a group title (a). Peptides associated with the same term are represented by a node. Node color represents the class that they belong. Mixed coloring means that the specific node belongs to multiple classes. Upregulated proteins are shown in red and downregulated are shown in blue. Edges show the association of the peptide with the terms. The thickness of the edge reflects the association significance. (b) Pie chart to indicate specific GO terms (% terms per group). **SI. Figure 8.** Gene Ontology and Functional enrichment analysis of DEPs in ABX-treated female AD (APPPS1-21-CD11br) mice using ClueGo and CluePedia plugins of Cytoscape. GO Terms (Biological Process, Cellular component, Molecular function, Immune pathway) associated with the identified proteins. The most significant parent or child term per functional group (kappa score ≥ 0.4) is shown in the functional grouped network as a group title (a). Peptides associated with the same term are represented by a node. Node color represents the class that they belong. Mixed coloring means that the specific node belongs to multiple classes. Upregulated proteins are shown in red and downregulated are shown in blue. Edges show the association of the peptide with the terms. The thickness of the edge reflects the association significance. (b) Pie chart to indicate specific GO terms (% terms per group). **SI. Figure 9.** Isolation and purification of microglia cells from 7-week-old WT-CD11br, vehicle or ABX-treated APPPS1-21-CD11br male and female mice (*n*=3). Following mechanical dissociation of cortical tissue from freshly perfused mouse brains and Percoll density centrifugation, mononuclear cells enriched for CD11b+ microglia were isolated via fluorescent activated cell sorting (a). (b) Representative flow cytometry gating strategy and co-staining of cells with CD11b-Alexa647 and CD45-Alexa488 antibodies for isolation of microglia CD11b^hi^-CD45^lo^. **SI. Figure 10.** Cecal analysis of APPPS1-21-CD11br transgenic mice treated with vehicle or ABX. A. (a) Representative images vehicle-treated male (M) and female (F) (top panel) and ABX-treated male (M) and female (F) (bottom panel). (b). One-way ANOVA analysis, Sidaks multiple comparison (adjusted *p*-val <0.0001) shows ABX-treatment resulted in significant increase in cecal weight compared to vehicle treated APPPS1-CD11br in both male and females. Heatmap to represent distinct cecal short-chain fatty acids (B), bile-acids (C) and Tryptophan (D) metabolites identified from metabolomic analysis of cecal content collected from WT-CD11br, vehicle or ABX-treated APPPS1-21-CD11br male and female mice. The metabolites with increased levels in ABX-treated mice are shown in bold. **Supplemental Table 1.** The list of top 10 upregulated and downregulated DEPs between vehicle-treated APPPS1-21-CD11br and WT-CD11br male and female mice. **Supplemental Table 2.** The list of top 10 upregulated and downregulated DEPs between ABX and vehicle-treated APPPS1-21-CD11br male and female mice. **Supplemental Table 3.** List of Primers used for RT-PCR and qPCR.

**Additional file 2. **

**Additional file 3. **

## Data Availability

All mass spectrometry data (raw files and MaxQuant search result files) are publicly available on ProteomeXchange repository (www.proteomexchange.org) with the identifier PXD035094.

## Abbreviations

AD: Alzheimer’s Disease
APP: Amyloid Precursor Protein
Aβ: Amyloid beta
ABX: Antibiotic
APOE4: Apolipoprotein 4
ATP: Adenosine Triphosphate
BBB: Blood Brain Barrier
CNS: Central Nervous System
DEP: Differentially expressed protein
EGFP: Enhanced Green Fluorescent Protein
ER: Endoplasmic Reticulum
FAD: Familial Alzheimer’s disease
FACS: Fluorescence activated cell sorting
GO: Gene Ontology
KEGG: Kyoto Encyclopedia of Genes and Genomes
LFQ: Label free quantification
LC-MSMS: Liquid chromatography coupled to tandem mass spectrometry
MCODE: Molecular Complex Detection
MGnD: Neurodegenerative Microglia
OXPHOS: Oxidative Phosphorylation
PS1: Presenilin
PPI: Protein-Protein Interaction
RNA: Ribonucleic Acid
ROS: Reactive Oxygen Species
SCFA: Short Chain Fatty Acid
STRING: Search Tool for the Retrieval of Interacting Genes/Proteins
TRAP: Translating ribosome affinity purification
