# Supplementary material for: Translational profiling identifies sex-specific metabolic and epigenetic reprogramming of cortical microglia/macrophages in APPPS1-21 mice with an antibiotic-perturbed-microbiome
Source: Mol Neurodegener. 2023 Dec 16;18:95. doi: 10.1186/s13024-023-00668-7 (PMC10725591; doi:10.1186/s13024-023-00668-7)
Supplement: Supplementary file 1 — Additional file 1: SI. Figure 1. Confirmation of APP/PS1 and FLAG-EGFP-Rpl10a transgenes in APPPS1-21-CD11br mice. A. Genomic DNA from APPPS1-21-CD11br mice was used for PCR amplification using primer set specific for APP, PS1 transgenes. APPPS1-21 mice serve as positive control. B. Schematic to show the binding sites of various primer sets to confirm the presence of FLAG-EGFP-Rpl10a transgene and the amplicon sizes. 1% Agarose gel to show the amplified products. SI. Figure 2. Characterization of CD11b+ cells from CD11br TRAP mice. Representative immunofluorescent images showing co-staining of CD11b with GFP and Iba I from brain sections of WT-CD11br and APPPS1-21-CD11br transgenic mice (A), microglia specific P2ry12 (magenta) and Tmem119 (red) markers colocalized with CD11b+ (green) macrophage/microglia cells from brain sections of 7-wks-old WT-CD11br and APPPS1-21-CD11br male mice (B), microglia specific P2ry12 (red) colocalized with CD11b+ (green) and 3D6 (magenta) stained for amyloid plaques in APPPS1-21-CD11br mice (C). (D) GFP (green) immunostaining colocalized with microglia marker P2ry12 (magenta) in brain sections of WT-CD11br, APPPS1-21-CD11br mice and APPPS1-21 that doesn’t show the expression of GFP was used as a control. The top panel of 2B-D shows low magnification and the selected microglia cells indicated in the box are shown at higher in the bottom panel. SI. Figure 3. Microglia specific transcripts from pull-down of cortical lysates of CD11br mice 7-week mice. Cortical lysates were used for immunoprecipitation of CD11b+ microglia/macrophage ribosomes from WT and APPPS1-21-CD11br male and female mice treated with vehicle or ABX and then for mRNA purification. Primer sets for Tmem119 (A) and P2ry12 (B) were used for RT-PCR analysis to confirm the pull-down of RNA from CD11b+ cells were abundant in microglia. Cortical lysates from APPPS1-21 and non-Tg mice were used as controls to indicate the specificity of pull-down. To account for the pulldown of C [file 13024_2023_668_MOESM1_ESM.docx]

**Translational profiling identifies sex-specific metabolic and epigenetic reprogramming of cortical microglia/macrophages in APPPS1-21 mice with an antibiotic-perturbed-microbiome**

Shabana M. Shaik^1^, Yajun Cao^1†^, Joseph V. Gogola^1†^, Hemraj B. Dodiya^1#^, Xulun Zhang^1#^, Hejer Boutej^2^, Weinong Han^1^, Jasna Kriz^2^ and Sangram S. Sisodia^1^*

^†^Equal contribution

^#^ Equal contribution

*Corresponding author

^1^Dept. of Neurobiology, The University of Chicago, Chicago, IL, USA,

^2^CERVO Brain Research Centre and Department of Psychiatry and Neuroscience^,^ Laval University, Québec, QC, Canada

**Supplemental Information**

M	1	2	3	4

1. No template control
2. APPPS1-21-CD11br
3. Negative control
4. APPPS1-21

750

500

300

**APP**

**PS1**

1. Negative Control
2. EGFP FP-EGFP RP
3. Flag FP-EGFP 3’
4. F/EGFP-Rpl10a FP- F/EGFP-Rpl10a RP

900 bp

300 bp

500 bp

1	2	3	4	M

A

B

**SI. Figure 1:** Confirmation of APP/PS1 and FLAG-EGFP-Rpl10a transgenes in APPPS1-21-CD11br mice. A. Genomic DNA from APPPS1-21-CD11br mice was used for PCR amplification using primer set specific for *APP*, *PS1* transgenes. APPPS1-21 mice serve as positive control. B. Schematic to show the binding sites of various primer sets to confirm the presence of FLAG-EGFP-Rpl10a transgene and the amplicon sizes. 1% Agarose gel to show the amplified products

**A**

**B**

**C**

**D**

**SI. Figure 2**: Characterization of CD11b+ cells from CD11br TRAP mice. Representative immunofluorescent images showing co-staining of CD11b with GFP and IBA I from brain sections of WT-CD11br and APPPS1-21-CD11br transgenic mice (A), microglia specific P2RY12 (magenta) and TMEM119 (red) markers colocalized with CD11b+ (green) macrophage/microglia cells from brain sections of 7-wks-old WT-CD11br and APPPS1-21-CD11br male mice (B), microglia specific P2RY12 (red) colocalized with CD11b+ (green) and 3D6 (magenta) stained for amyloid plaques in APPPS1-21-CD11br mice (C). (D) GFP (green) immunostaining colocalized with microglia marker P2RY12 (magenta) in brain sections of WT-CD11br, APPPS1-21-CD11br mice and APPPS1-21 that doesn’t show the expression of GFP was used as a control. The top panel of 2B-D shows low magnification and the selected microglia cells indicated in the box are shown at higher in the bottom panel.

**SI. Figure 3:** Microglia specific transcripts from pull-down of cortical lysates of CD11br mice 7-week mice. Cortical lysates were used for immunoprecipitation of CD11b+ microglia/macrophage ribosomes from WT and APPPS1-21-CD11br male and female mice treated with vehicle or ABX and then for mRNA purification.

Primer sets for *Tmem119* (A) and *P2ry12* (B) were used for RT-PCR analysis to confirm the pull-down of RNA from CD11b+ cells were abundant in microglia. Cortical lysates from APPPS1-21 and non-Tg mice were used as controls to indicate the specificity of pull-down. To account for the pulldown of CD11b expressing macrophages, following the synthesis of cDNA, primers specific for *Hexb*, a bonafide microglia marker and *CD163*, a selective marker of perivascular macrophages were used for RT-PCR and the amplicons were resolved on 1% Agarose gel (C, a). The ΔCt (Ct [Gene]-Ct [Gapdh]) values from qPCR analysis using the same primer sets to indicate the pulldown of CD11b+ cells were enriched for microglia (C, b).

**A**

**B**

**SI. Figure 4: A.** Number of protein quantifications for each group comparison identified by LFQ**. B.** Venn diagram representing the number of proteins found in all replicates of each group of male mice (RM, TMV, TMA) and female mice (RF, TFV, TFA).

**A**

**B**

**SI. Figure 5**: Gene Ontology and functional enrichment analysis of DEPs in vehicle-treated male AD (APPPS1-21-CD11br) mice using ClueGo and CluePedia plugins of Cytoscape. GO Terms (Biological Process, Cellular component, Molecular function, Immune pathway) associated with the identified proteins. The most significant parent or child term per functional group (kappa score ≥ 0.4) is shown in the functional grouped network as a group title (**a**). Peptides associated with the same term are represented by a node. Node color represents the class that they belong. Mixed coloring means that the specific node belongs to multiple classes. Upregulated proteins are shown in red and downregulated are shown in blue. Edges show the association of the peptide with the terms. The thickness of the edge reflects the association significance. **(b)** Pie chart to indicate specific GO terms (% terms per group).

**A**

**B**

**SI. Figure 6**: Gene Ontology and functional enrichment analysis of DEPs in ABX-treated male AD (APPPS1-21-CD11br) mice using ClueGo and CluePedia plugins of Cytoscape. GO Terms (Biological Process, Cellular component, Molecular function, Immune pathway) associated with the identified proteins. The most significant parent or child term per functional group (kappa score ≥ 0.4) is shown in the functional grouped network as a group title (a). Peptides associated with the same term are represented by a node. Node color represents the class that they belong. Mixed coloring means that the specific node belongs to multiple classes. Upregulated proteins are shown in red and downregulated are shown in blue. Edges show the association of the peptide with the terms. The thickness of the edge reflects the association significance. **(b)** Pie chart to indicate specific GO terms (% terms per group).

**A**

**B**

**SI. Figure 7**: Gene Ontology and Functional enrichment analysis of DEPs in vehicle-treated female AD (APPPS1-21-CD11br) mice using ClueGo and CluePedia plugins of Cytoscape. GO Terms (Biological Process, Cellular component, Molecular function, Immune pathway) associated with the identified proteins. The most significant parent or child term per functional group (kappa score ≥ 0.4) is shown in the functional grouped network as a group title (a). Peptides associated with the same term are represented by a node. Node color represents the class that they belong. Mixed coloring means that the specific node belongs to multiple classes. Upregulated proteins are shown in red and downregulated are shown in blue. Edges show the association of the peptide with the terms. The thickness of the edge reflects the association significance. **(b)** Pie chart to indicate specific GO terms (% terms per group).

**A**

**B**

**SI. Figure 8**: Gene Ontology and Functional enrichment analysis of DEPs in ABX-treated female AD (APPPS1-21-CD11br) mice using ClueGo and CluePedia plugins of Cytoscape. GO Terms (Biological Process, Cellular component, Molecular function, Immune pathway) associated with the identified proteins. The most significant parent or child term per functional group (kappa score ≥ 0.4) is shown in the functional grouped network as a group title (a). Peptides associated with the same term are represented by a node. Node color represents the class that they belong. Mixed coloring means that the specific node belongs to multiple classes. Upregulated proteins are shown in red and downregulated are shown in blue. Edges show the association of the peptide with the terms. The thickness of the edge reflects the association significance. **(b)** Pie chart to indicate specific GO terms (% terms per group).

**SI. Figure 9:** Isolation and purification of microglia cells from 7-week-old WT-CD11br, vehicle or ABX-treated APPPS1-21-CD11br male and female mice (n=3).

Following mechanical dissociation of cortical tissue from freshly perfused mouse brains and Percoll density centrifugation, mononuclear cells enriched for CD11b+ microglia were isolated via fluorescent activated cell sorting (**a**). (**b**) Representative flow cytometry gating strategy and co-staining of cells with CD11b-Alexa647 and CD45-Alexa488 antibodies for isolation of microglia CD11b^hi^-CD45^lo^.

**A**

a

b

**A**

**B**

**B**

**C**

**D**

**SI. Figure 10**: Cecal analysis of APPPS1-21-CD11br transgenic mice treated with vehicle or ABX. **A**. (a) Representative images vehicle-treated male (M) and female (F) (top panel) and ABX-treated male (M) and female (F) (bottom panel). **(b)**. One-way ANOVA analysis, Sidaks multiple comparison (adjusted p-val <0.0001) shows ABX-treatment resulted in significant increase in cecal weight compared to vehicle treated APPPS1-CD11br in both male and females. Heatmap to represent distinct cecal short-chain fatty acids (**B**), bile-acids (**C**) and Tryptophan (**D**) metabolites identified from metabolomic analysis of cecal content collected from WT-CD11br, vehicle or ABX-treated APPPS1-21-CD11br male and female mice. The metabolites with increased levels in ABX-treated mice are shown in bold.

| **Sex** | **Upregulated**  **DEP in AD** | **Log2**  **Fold-change** | **Downregulated**  **DEP in AD** | **Log2**  **Fold-change** |
| --- | --- | --- | --- | --- |
| Male | Rps26  Rpl10  Prkce  Pitpnm1  Slc25a12  Pitpna  Rock1  Spag9  Prkca  Atp2a2 | 4.3  3.2  2.84  2.73  2.5  2.36  2.24  2.19  2.18  2.10 | Tmp1  Vapb  Wdr7  Vdac2  Cpne8  Vdac1  Fabp5  Tmsb4x  Dctn5  Vdac3 | 3.51  3.11  2.89  2.87  2.56  2.5  2.5  2.5  2.5  2.5 |
| Female | Top1  Ndufs4  Sirpa  Chl1  Cadm3  Ptn  Cap2  Sub1  Cbln1  Rab16 | 6.10  4.69  4.56  4.35  4.08  4.06  3.98  3.98  3.85  3.82 | Rps9  Rps5  Rps8  Rps18  Rps16  Rps2  Pa2g4  Rpl38  Rps13  Rps14 | 12.06  11.62  10.59  10.37  10.07  10.01  9.93  9.92  9.91  9.54 |

**Supplemental Table 1:** The list of top 10 upregulated and downregulated DEPs between vehicle-treated APPPS1-21-CD11br and WT-CD11br male and female mice.

| **Sex** | **Upregulated**  **DEP in ABX-treatment** | **Log2**  **Fold-change** | **Downregulated**  **DEP in ABX-treatment** | **Log2**  **Fold-change** |
| --- | --- | --- | --- | --- |
| Male | Camk2n1  Gpd2  Sirpa  Adam22  Rpl1p1  Rhog  Slc8a2  Ankfy1  Pdcd6  Cadm3 | 4.83  4.67  4.63  4.41  4.29  4.25  4.04  3.94  3.85  3.84 | Hist1h1c  Hist1h1e  Basp1  Clns1a  Gipc1  Eif4b  Hist1h3a/b  H3f3a  Cox6c  Gpd1 | 6.86  5.54  5.33  5.31  5.08  5.0  4.95  4.95  4.88  4.59 |
| Female | Rps5  Rps9/17  Ppm1b  Rpl38  Isoc2a  Rpl10/10l/Rplp0  Rps19/Rpl8/Rps15a  Rpl22/12/Rps28  Rps2/24  Tmod1 | 8.09  7.74  7.19  7.06  7.01  6.9  6.6  6.4  6.2  6.0 | Mt-Atp8  Ighg1  Plp1  Snrnp40  Erh  Cox4i1  Ndufa13  Astn1  Vdac1 | 4.59  4.39  4.1  4.06  3.86  3.69  3.47  3.09  2.84 |

**Supplemental Table 2:** The list of top 10 upregulated and downregulated DEPs between ABX and vehicle-treated APPPS1-21-CD11br male and female mice.

| Primer | Primer seq (5’-3’) |
| --- | --- |
| EGFP FP  EGFP RP  F/EGFP-Rpl10a FP  F/EGFP-Rpl10a FP  Gapdh FP  Gapdh RP  Iba I FP  Iba I RP  Tmem119 FP  Tmem119 RP  CX3CR1 FP  CX3CR1 FP  P2ry12 FP  P2ry12 RP  Trem2 FP  Trem2 RP  GFAP FP  GFAP RP  Tubb3 FP  Tubb3 RP  Cldn11 FP  Cldn11 RP  Atp6v0d1 FP  Atp6v0d1 RP  Camk2n1 FP  Camk2n1 RP  Calm1 FP  Calm1 RP  Sirpa FP  Sirpa RP  Bdh1 FP  Bdh1 RP  Hexb FP  Hexb RP  Gapdh FP  Gapdh RP | AGTTCATCTGCACCACCGGC  CGGCCATGATATAGACGTTG  AAGACGATGACGACAAGATGG  GGTGTCGCGTGAGACTTT  AACAGCAACTCCCACTCTTC  CCTGTTGCTGTAGCCGTATT  AGCCTGAGGAGATTTCAACAGAA  CCTCAGACGCTGGTTGTCTT  CACCCAGAGCTGGTTCCATA  GAGTGACACAGAGTAGGCCA  AGTCAACATCCTGGGCTTCG  AGGATGAGTCTGACGGCTCT  CACGGACACTTTCCCGTATCC  GCCTTGAGTGTTTCTGTAGGGT  GACCTCTCCACCAGTTTCTCC  TACATGACACCCTCAAGGACTG  CACCTACAGGAAATTGCTGGAGG  CCACGATGTTCCTCTTGAGGTG  TGAGGCCTCCTCTCACAAGT  TGCAGGCAGTCACAATTCTC  CAGGCTTGTAGAGCCCTCAT  GTGGGCACATACAGGAAACC  GCATCTCAGAGCAGGACCTTGA  GGATAGGACACATGGCATCAGC  ATGGCTCCTTCTGGATTCCCTG  TCCAGTTCCGAGAGTAAGGCAG  GCACCATCACAACCAAGGAACTG  ACTCTGGGAAGTCAATGGTGCC  TCATCTGCGAGGTAGCCCACAT  ACTGTTGGGTGACCTTCACGGT  AGGCTGTGACTCTGGATTTGGG  CTGGATGGTTCTCAGTCGGTCA  GCTGTTGGTGAGAGACTCTGGA  GAGGTTGTGCAGCTATTCCACG  CATCACTGCCACCCAGAAGACTG  ATGCCAGTGAGCTTCCCGTTCAG |

**Supplemental Table 3:** List of Primers used for RT-PCR and qPCR
